# Levels of DNA methylation and transcript accumulation in leaves of transgenic maize varieties

**DOI:** 10.1186/s12302-016-0097-2

**Published:** 2016-11-23

**Authors:** Vinicius Vilperte, Sarah Zanon Agapito-Tenfen, Odd-Gunnar Wikmark, Rubens Onofre Nodari

**Affiliations:** 1 Department of Crop Science, Federal University of Santa Catarina, Florianópolis, Santa Catarina Brazil; 2 GenØk - Centre for Biosafety, Tromsø, Norway; 3 Unit for Environmental Science and Management, Potchefstroom Campus, North West University, Potchefstroom, South Africa; 4 Institute for Plant Genetics, Faculty of Natural Sciences, Leibniz University of Hannover, Hannover, Germany

**Keywords:** Genetically modified organism, Epigenetics, DNA methylation, Stacked GMO, Risk assessment, Genetic stability, *Zea mays*

## Abstract

**Background:**

Prior to their release in the environment, transgenic crops are examined for their health and environmental safety. In addition, transgene expression needs to be consistent in order to express the introduced trait (e.g. insecticidal and/or herbicide tolerance). Moreover, data on expression levels for GM events are usually required for approval, but these are rarely disclosed or they are considered insufficient. On the other hand, biosafety regulators do not consider epigenetic regulation (e.g. DNA methylation, ncRNAs and histone modifications), which are broadly known to affect gene expression, within their risk assessment analyses. Here we report the results of a DNA methylation (bisulfite sequencing) and transgene transcript accumulation (RT-qPCR) analysis of four Bt-expressing single transgenic maize hybrids, under different genetic backgrounds, and a stacked transgenic hybrid expressing both insecticidal and herbicide tolerance traits.

**Results:**

Our results showed differences in cytosine methylation levels in the FMV promoter and *cry2Ab2* transgene of the four Bt-expressing hybrid varieties. The comparison between single and stacked hybrids under the same genetic background showed differences in the 35S promoter sequence. The results of transgene transcript accumulation levels showed differences in both *cry1A.105* and *cry2Ab2* transgenes among the four Bt-expressing hybrid varieties. The comparison between single and stacked hybrids showed difference for the *cry2Ab2* transgene only.

**Conclusions:**

Overall, our results show differences in DNA methylation patterns in all varieties, as well as in transgene transcript accumulation levels. Although the detection of changes in DNA methylation and transgenic accumulation levels does not present a safety issue per se, it demonstrates the need for additional studies that focus on detecting possible safety implications of such changes.

**Electronic supplementary material:**

The online version of this article (doi:10.1186/s12302-016-0097-2) contains supplementary material, which is available to authorized users.

## Background

The Cartagena Protocol on biosafety defines living modified organism (LMO), also known as genetically modified organism (GMO), as “*any living organism that possesses a novel combination of genetic material obtained through the use of modern biotechnology*” [1]. It also defines modern biotechnology as the application of “in vitro *nucleic acid techniques, including recombinant deoxyribonucleic acid (DNA) and direct injection of nucleic acid into cells or organelles, or ii) fusion of cells beyond the taxonomic family and that are not techniques used in traditional breeding and selection*”. This protocol acts as a regulatory document for assessing the biosafety of GMOs in an international level. The use of agricultural GMOs has been growing steadily over the last decade [2], which shows the need for adequate regulation of its use.

Domestic and international GMO regulations, such as The Cartagena Protocol, provide guidance regarding GMO risk assessment in order to ensure, among other safety aspects, the genetic stability of a transgene. Although there is no agreed operational definition for the stability concept among regulators, these measures may include the identification of any novel genotypic and phenotypic characteristics associated with the GMO that may have adverse effects on biological diversity, as well as information regarding the genetic characteristics of the inserted nucleic acid and the function it specifies, and/or characteristics of the modification introduced [1].

Plant genetic engineering was mainly achieved through *Agrobacterium*-mediated transformation, which takes advantage of the natural ability of the soil bacterium *Agrobacterium tumefaciens* to transfer a segment of its DNA, the T-DNA, into the host plant genome [3], or through particle bombardment (biobalistic or gene gun), which relies on the delivery of gold particles coated with the DNA to be inserted into the plant nuclear genome [4]. After plants have been transformed, regeneration through in vitro culture can cause rearrangements in the transgene sequence or even in the host genome [5], sometimes showing extensive genomic variations, especially epigenetic changes, in the process of micro propagation, which may or may not result in phenotypic changes [6], but once transgenic plants have been generated, it is assumed that the transgene is stable and mutations occur at the same rates as endogenous genes [7].

Routine genetic stability analyses performed by GMO developers usually rely on protein quantification (ELISA—*Enzyme*-*Linked Immuno*-*Sorbent Assay*) and transgene integrity (Southern blot) [8, 9]. Independent studies have also assessed genetic stability of GMO events not only with different approaches (e.g. cytogenetics and Southern Blot) [10, 11] but also with more rigorous criterions, such as seasonal and tissue-specific ELISA analysis [12] and RNA expression using Northern blot technique [11]. In addition, studies have detected transgene rearrangements, such as sequence deletions [13–15] and nucleotide addition of undesired fragments into the transgene sequence [16], and also, alterations in mRNA expression levels were also observed [17]. However, changes in DNA sequences are not the only source of alteration in transgene expression. Epigenetics—defined as the study of molecular mechanisms involved in hereditable changes—are able to regulate gene expression without changing the DNA sequence per se [18]. These variations are often associated with DNA methylation, histone modifications and non-coding RNAs (ncRNAs), which can lead to phenotypic variation and transgene silencing [19]. Both ncRNAs and histone modifications have been linked to RNAi (RNA interference), PTGS (Post-Transcriptional Gene Silencing) and TGS (Transcriptional Gene Silencing) pathways [20], and are associated with up-regulation of gene transcription or with transcription repression [21, 22].

Cytosine methylation is an epigenetic regulatory mechanism that is able to control gene expression by inhibiting protein binding to DNA and by changing chromatin structure [23]. Plant DNA sequences are known to be highly methylated, mostly 5-methylcytosine (m^5^C), which is located mainly in symmetrical CG sites. However, the high amount of m^5^C found in some plant species suggested that methylation is not restricted to the CG sequence context but also methylated in CHG (where H is A or T) and CHH sites (where H is A, C or T) [24–26]. Investigations of cytosine methylation patterns have been conducted in transgenic models such as *Arabidopsis thaliana* [27–29] and *Petunia hybrida* [30, 31]. Methylation changes have been also studied in maize, targeting endogenous genes and transposable elements [32–34]. To date, La Paz et al. [6] conducted the only study regarding epigenetic aspects of commercialized GM crops, in which the authors analysed the cytosine methylation levels of different Bt-expressing (MON810) varieties.

Therefore, in order to contribute to better understanding of epigenetic mechanisms that may impact transgene expression of GMOs, the aim of this study was to investigate the epigenetic profiles of the transgenic cassette of a double-Bt-expressing transgenic maize event (MON-89Ø34-3), as well as the transcription expression of the inserted traits. We have analysed the levels of cytosine methylation by bisulfite-sequencing technique, we have verified the transgene transcript accumulation by RT-qPCR and compared single and stacked GM maize hybrid varieties containing the MON-89Ø34-3 event. In addition, this manuscript also aims to provide relevant information and insight for reliable risk assessments of GMOs.

## Methods

**Fig. 1:**
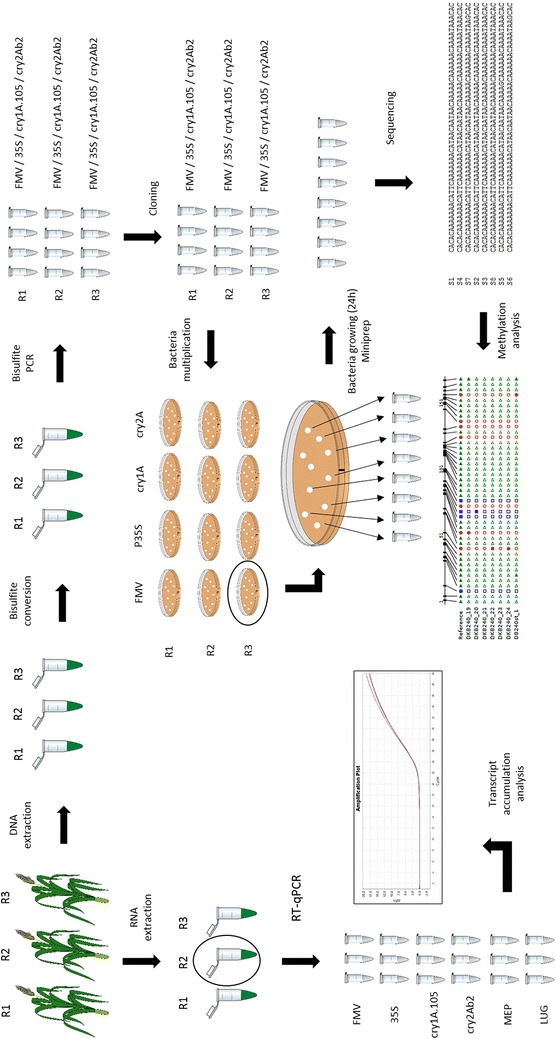
Schematic overview of the experimental design, sampling strategy and analytical approach used in the study. Leaf samples of single and stacked GM events (MON-89Ø34-3 and MON-89Ø34-3 × MON-ØØ6Ø3-6, respectively) were used for DNA methylation and transgene transcript accumulation analysis. Three biological replicates were analysed for each of the GM varieties. Four regions of the transgene cassette (FMV promoter, *cry1A.105* transgene, 35S promoter and *cry2Ab2* transgene) were chosen for DNA methylation analysis. The accumulation of *cry1A.105* and *cry2Ab2* transgenes was quantified by RT-qPCR

A schematic overview of our experimental design, sampling strategy and analytical approach is provided in Fig. 1.

### Plant material and growth conditions

Five maize varieties were used in this study. Four of them are considered single-event varieties because they contain only one transgenic event with resistance to some lepidopteran species: DKB240PRO and DKB350PRO (unique identifier MON-89Ø34-3 from Monsanto Company, resistance to some lepidopteran species, distributed by Dekalb) and AG8041PRO and AG9045PRO (unique identifier MON-89Ø34-3 from Monsanto Company, resistance to some lepidopteran species, distributed by Sementes Agroceres). The DKB240PRO2 (unique identifier MON-89Ø34-3 × MON-ØØ6Ø3-6 from Monsanto Company, stacked event resistant to some lepidopteran species and a class of herbicide, Dekalb) is considered a stacked event because it contains two transgenic events. The combination of the two transgenic events in a single hybrid was obtained by traditional crossing. These are named in this study as DKB240, DKB350, AG8041, AG9045 and DKB240-ST, respectively (Table 1). The used varieties are hybrid progenies of the single cross between maternal endogamous line “A” with the paternal endogamous line “B”. Thus, the hybrid variety seeds used have high genetic similarity (all seeds are AB genotype). All these five commercial varieties were produced by the aforementioned company and are commonly found in the seed market in southern Brazil.

**Table 1:** Transgenic commercial maize varieties used in this study

Commercial name	GM event	Transgenes	Trait	# of biological replicates	Labelled in this study
DKB240PRO	MON-89Ø34-3	cry1A.105/cry2Ab2	IR	3	DKB240
DKB350PRO	MON-89Ø34-3	cry1A.105/cry2Ab2	IR	3	DKB350
AG8041PRO	MON-89Ø34-3	cry1A.105/cry2Ab2	IR	3	AG8041
AG9045PRO	MON-89Ø34-3	cry1A.105/cry2Ab2	IR	3	AG9045
		cry1A.105/cry2Ab2			
	MON-89Ø34-3				
DKB240PRO2	x	x	IR/HT	3	DKB240-ST
	MON-ØØ6Ø3-6	epsps/epsps			

*IR* insect-resistant, *HT* herbicide-tolerant

The transformation events MON-89Ø34-3 and MON-89Ø34-3 × MON-ØØ6Ø3-6 were approved for commercial use in Brazil in 2009 [35] and 2010 [36], respectively. The MON-89Ø34-3 event expresses two insecticidal proteins (*cry1A.105* and *cry2Ab2* proteins derived from *Bacillus thuringiensis*, which are active against certain lepidopteran insect species) and the MON-ØØ6Ø3-6 event expresses the enzyme *CP4*-*epsps* (5-enolpyruvylshikimate-3-phosphate synthase) that confers tolerance against glyphosate-based herbicides.

The seeds from all five varieties were grown side-by-side in a controlled environment set to 16 h light period and 25 °C (±2 °C). Seedlings were germinated and grown in 3 L plastic pots with Plantmax HT substrate (Buschle & Lepper S.A.) and watered daily. No pesticide or fertilizer was applied. Ten plants were grown in controlled climate out of which leaf samples from 3 random plants sampled per maize variety (genotype). Therefore, each variety contained 3 biological replicates that were used for the experiment. In order to standardize the sampling, the third and fourth leaves, starting from the bottom, were sampled for each plant and kept at −80 °C until RNA and DNA extraction.

### Bisulfite treatment of genomic DNA

Genomic DNA from three biological replicates for each variety was isolated from 100 mg of frozen leaf tissue using the column-based NucleoSpin^®^ Plant II (Macherey–Nagel GmbH & Co. KG, Germany) extraction kit and further quantified in a NanoDrop 2000c spectrophotometer (Thermo Scientific, Wilmington, USA). Subsequently, 200 ng of isolated DNA was submitted to bisulfite treatment in order to convert non-methylated cytosines into uracil. The conversion was performed using the EpiTect^®^ Bisulfite Kit (Qiagen, Hilden, Germany) following manufacturer’s recommendations.

### Bisulfite PCR and fragment purification

Four regions of the MON-89Ø34-3 transgenic insert were chosen to analyse the patterns of methylation: one in the e35S promoter, one in the *cry1A.105* transgene, one in the FMV promoter and one in the *cry2Ab2* transgene. Four modified pairs of primers, designed to amplify the converted DNA, were designed using the MethPrimer software [37] (Table 2). The size and the amount of cytosine types—CG, CHG (where H is nitrogenous base A or T) and CHH (where H is a nitrogenous base different from G)—of each fragment are shown in Table 3. In order to differentiate the 35S promoter of the *35S:cry1A.105* construct from the *35S:EPSPS* construct, the BS35S-F primer was designed to anneal in a sequence containing 8 SNPs between the promoter regions (Additional file 1). Therefore, only the 35S promoter regions from the *cry1A.105* transgene are expected to be amplified.

**Table 2:** Bisulfite primers used to amplify four DNA regions after bisulfite conversion

Primer name	Sequence	GM region	*Ta* (°C)
BS35S-F	5′-TTATTAAAAGGATAGTAGAAAAGG-3′	P-e35S	51
BS35S-R	5′-AAAACCTCCCTTAAATCTTATAAT-3′		
BScry1A.105-F	5′-ATTATTTGGGGTATTTTTGGTTTTTT-3′	*cry1A.105*	55
BScry1A.105-R	5′-AAACCCCACCTCTAACCAAACA-3′		
BSFMV-F	5′-GTTTGTGGGGATTAGATAAAAAA-3′	P-FMV	50
BSFMV-R	5′-CACACAAAAAAACATTCAAAAAAA-3′		
BScry2Ab2-F	5′-TTTATGTTTTGTTTGTTGTTAGAGTGA-3′	*cry2Ab2*	51
BScry2Ab2-R	5′-ACCAAAAATATTCCTAATAAAATAAT-3′		

*Ta* annealing temperature, *F* forward primer, *R* reverse primer

**Table 3:** Fragment size and number of cytosine types for each of the transgenic fragments analysed

Fragment of the transgene construct	Cytosine type			Fragment size (bp)
	CG	CHG	CHH	
FMV promoter	12	13	61	340
*cry1A.105* gene	24	32	72	365
35S promoter	11	9	84	388
*cry2Ab2* gene	27	23	56	290

*CHG* H is nitrogenous base A or T, *CHH* H is a nitrogenous base different from G

After bisulfite treatment, the bisulfite-converted DNA was used to PCR amplify the four regions in each of the three replicates for each variety. The PCR conditions were 1× PfuTurbo Cx reaction buffer, 0.2 mM of each dNTP, 0.5 µM of forward and reverse primers, 1 U of PfuTurbo Cx hotstart DNA polymerase (Agilent Technologies, California, USA) and 60 ng of converted DNA. The steps used for the amplification were 95 °C for 3 min; 35 cycles of 95 °C for 30 s annealing temperature (specific for each primer) for 30 s, 65 °C for 2 min and 65 °C for 10 min. The reactions were carried out in a S1000™ Thermal Cycler (BioRad, California, USA).

The PCR products were stained with GelRed™ (Uniscience, Florida, USA) and resolved in 1% (w/v) agarose gel in horizontal electrophoresis for 90 min at 100 V. Bands were excised from the gel and purified using the NucleoSpin^®^ Gel and PCR Clean-up (Macherey–Nagel) following manufacturer’s recommendations.

### Cloning

Each of the four regions from the three replicates of each variety were cloned in the pCR II-Blunt-TOPO vector using the Zero Blunt^®^ TOPO^®^ PCR Cloning Kit (Life Technologies, California, USA), following manufacturer’s recommendations, and transformed in chemically competent *Escherichia coli* cells (TOP10 strain). Briefly, 2 μL of the cloning reaction were added to *E. coli* cells, thoroughly agitated and incubated in ice for 10 min. Following, the cells were submitted to thermal shock at 42 °C for 30 s and immediately transferred to ice. 250 μL of SOC medium (provided with the kit) were added to the cells and horizontally agitated (200 rpm) for 1 h at 37 °C. Lastly, 40 μL of the *E. coli* + SOC medium solution were added to a petri dish containing solid Luria–Bertani (LB) medium [38] supplemented with 50 μg/mL of kanamycin antibiotic. The plates were incubated overnight at 37 °C for the bacteria colonies to grow.

### Cell multiplication and Miniprep

Previously cultivated *E. coli* cells were multiplied in order to increase the number of colonies to be cloned. Eight random bacteria colonies (eight different clones) for each fragment were selected and transferred to a bacteria-growing plate (96-wells) containing liquid LB medium (supplemented with 50 μg/mL of kanamycin antibiotic). The plate was sealed and agitated at 37 °C for 22 h.

After multiplication, the plate was centrifuged for 6 min (2000*g*) at room temperature for pellet formation. The plate was inverted to remove the supernatant. The Miniprep was performed using the NucleoSpin Plasmid kit^®^ (Macherey–Nagel) following manufacturer’s recommendations.

### Sequencing

The cloned fragments were sequenced using the M13 reverse universal primer (5′-CAGGAAACAGCTATGAC-3′) by chain-terminator technique [39] using BigDye Terminator v3.1 Cycle Sequencing Kit (Applied Biosystems^®^, Singapore, Singapore). The sequencing reactions were performed in a volume of 20 μL containing 1 μL of BigDye v3.1, 3 μL of BigDye v3.1 reaction buffer, 3.5 pmol of the M13 reverse universal primer and 140 ng of the plasmid containing the fragments. The steps used for the amplification were 96 °C for 5 min, 25 cycles of 96 °C for 10 s, 50 °C for 5 s and 60 °C for 4 min. The reactions were carried out in a S1000™ Thermal Cycler (BioRad). Purification of sequencing reactions and capillary electrophoresis were performed in the DNA sequencing core facility at the University Hospital of North Norway (Universitetssykehuset Nord-Norge, Tromsø, Norway) using a Genetic Analyzer 3130xl (Applied Biosystems^®^).

### Relative quantification analysis of transgene transcripts

Total RNA was extracted from leaf tissues using the column-based RNeasy Plant Mini Kit (Qiagen). Reverse-transcription quantitative PCR (RT-qPCR) assay was adapted from previously developed assays for the specific detection of MON-89Ø34-3 × MON-ØØ6Ø3-6 transgenes [40] to hydrolysis ZEN—Iowa Black^®^ Fluorescent Quencher (ZEN/IBFQ) probe chemistry (Integrated DNA Technologies, INC Iowa, USA). Following quantification, cDNA was synthesized, and amplification of each target gene was performed using the QuantiTect Probe RT-PCR Kit (Qiagen) according to the manufacturer’s instructions.

RT-qPCR experiment was carried out in technical triplicates using StepOne™ Real-Time PCR System (Applied Biosystems). Each 20 μL reaction volume comprised 10 μM of each primer and probe and 50 ng of total RNA from each sample. The amplification efficiency was obtained from relative standard curves provided for each primer and calculated according to Pfaffl equations [41]. The choice of the endogenous reference genes and the selection of the two best genes were based on the previous work of Agapito-Tenfen et al. [42], using NormFinder (Molecular Diagnostic Laboratory, Aarhus University Hospital Skejby, Denmark) statistical algorithms [43].

The *leunig* and *membrane protein PB1A10.07c* genes were used to normalize *cry1A.105* and *cry2Ab2* mRNA data due to their best stability value (SV for best combination of two genes 0.025, Additional file 2). Conventional samples were also analysed in order to check for PCR and/or seed contaminants. Primer and probe sequences used, as well as Genebank ID of target genes, are provided in Table 4. The primers and probes were assessed for their specificity with respect to known splice variants and single-nucleotide polymorphism positions documented in transcript and single-nucleotide polymorphism databases. The normalized relative quantity (NRQ) was calculated for both single and stacked transgenic events samples relative to one of the three DKB240 samples according to the Pfaffl equations [41].

**Table 4:** Description of reference genes and transgenes used for quantification of transcripts and their primer sequences

Primer name	Gene product	Genbank accession no.	Primer sequence
MEP	Membrane protein PB1A10.07c	GRMZM2G018103_T01	F-GTACTCGGCAATGCTCTTGA
			P-AACTTCGGTTGGTGAGAGCGGAAA
			R-CAATCCTGACCCAGACAGATG
LUG	Leunig	GRMZM2G425377_T01	F-GGGACATAAGGGAGAAGAACAC
			P-TTCCCTGTAGCACTGGATGATGCC
			R-TCATGGCTTACTGAGGCAAC
Cry1A.105	Cry1A.105 protein	FV532179/FB707509	F-GACGTGGAGGAACAGAACAA
			P-TTGTGCCTGAGTGGGAAGCTGAA
			R-CCTCTACCTGGACAGACTCTAA
Cry2Ab2	Cry2Ab2 protein	FV532179/AR260587	F-GCGACTACCTGAAGAACTACAC
			P-CAACACCTACCAGTCGGCCTTCAA
			R-TGTCGTGAAGCCTCGTATTG

*F* forward primer, *R* reverse primer, *P* probe

### Statistical analysis

For DNA methylation analysis, quality of the sequences were accessed using the Sequence Scanner 2 software (Applied Biosystems^®^), DNA methylation levels (%) in CG, CHG and CHH cytosine types were assessed using the CyMATE web tool [44], and statistical analyses were performed with R language and statistical environment [45] using in-house scripts. To calculate the percentage (%) of methylated cytosines in each fragment, the number of methylated cytosines was divided by the number of total cytosines (available in Table 2) for each analysed fragment sequence. Wilcoxon-Mann–Whitney test for non-parametric data (*p* < 0.05) was used for the comparative analysis of the single event (MON-89Ø34-3) versus stacked event (MON-89Ø34-3 × MON-ØØ6Ø3-6) and Kruskal–Wallis test for non-parametric data (*p* < 0.05) was used for the comparative analysis among the single events (MON-89Ø34-3).

For the transcript quantification, normalized gene expression data were obtained using the Pfaffl method for efficiency correction [41], which is based on a mathematical model for relative quantification in real-time PCR where the efficiency of each primer is considered as a normalizer factor from the results. Cq average from each technical replicate was calculated for each biological replicate and used to make a statistical comparison of varieties based on the standard deviation. Due to non-normal distribution, the fold change data were log10 transformed before statistical tests. The fold change means obtained for the single varieties, as well as for single versus stacked GM event, were compared using ANOVA/Tukey-test and *T* test, respectively, at *p* < 0.05 (R language) [45]. Information on real-time data for this study has followed guidelines from the minimum information for publication of quantitative real-time PCR experiments [46].

## Results

### Cytosine methylation levels in Bt-expressing transgenic maize

MON-89Ø34-3 is a genetically modified maize containing two transgenes: one chimeric cry1 delta-endotoxin (CRY1A.105) is inserted under the regulation of a Cauliflower Mosaic Virus promoter (CaMV 35S) and a Figwort Mosaic Virus (FMV) 35S promoter driving cry2Ab delta-endotoxin (CRY2AB2) expression. In order to study the DNA methylation levels, three types of cytosines—CG, CHG and CHH—were analysed in four different regions of the transgenic insert: (i) FMV promoter; (ii) 35S promoter; (iii) *cry1A.105* transgene; and (iv) *cry2Ab2* transgene, in two experimental setups.

**Fig. 2:**
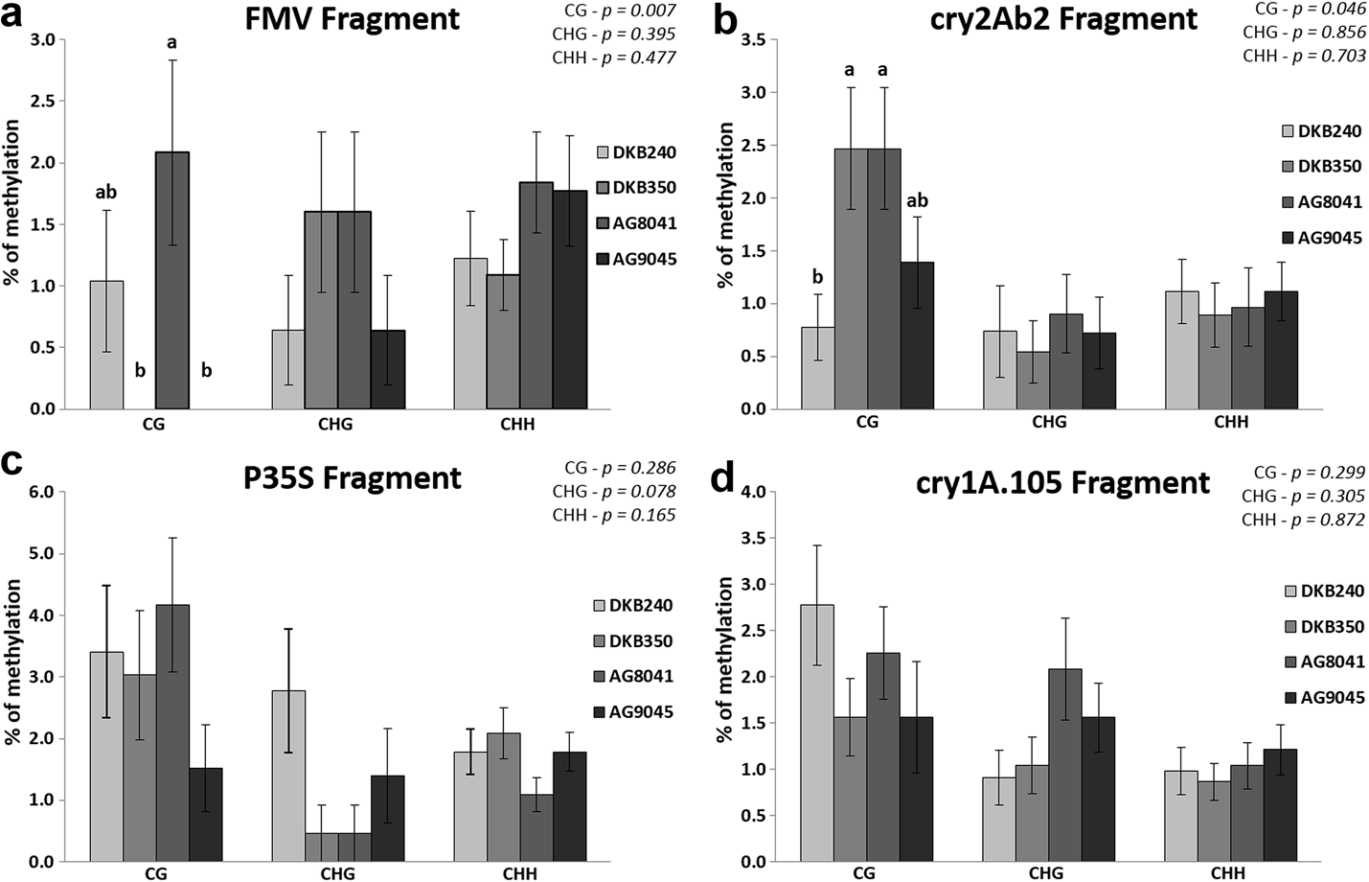
Cytosine methylation levels among MON-89Ø34-3 maize varieties in four regions of the transgenes. The levels of cytosine methylation were measured in CG, CHG and CHH residues. **a** FMV promoter region; **b** *cry2Ab2* transgene region; **c** 35S promoter region; **d** *cry1A.105* transgene region. *Vertical bars* indicate standard errors. Means followed by *different letters* in the same cytosine type are significantly different according to the Kruskal–Wallis test (*p* < 0.05). Exact *p* values for the statistical test of each of the cytosine comparisons are shown on the *right upper* corner

The first experiment setup analysed the transgenic regions among four different commercial available varieties (DKB350, AG9045, AG8041 and DKB240) containing the MON-89Ø34-3 event under different genetic backgrounds. The analysis of the FMV promoter region showed significant differences between the varieties for the CG cytosine type, according to the Kruskal–Wallis test for non-parametric data (*p* = 0.007), while there was no statistical difference in methylation patters among CHG and CHH types. The AG8041 variety showed 2.08% of methylated CG, DKB240 showed 1.04% and DKB350 and AG9045 showed no methylation in this region (Fig. 2a).

The *cry2Ab2* region also showed significant differences, according to the Kruskal–Wallis test (*p* = 0.046), for the CG residues, but not for the other cytosine types. DKB350 and AG8041 varieties showed 2.46% of methylated cytosines, AG9045 showed 1.38% and DKB240 showed 0.77% (Fig. 2b). The *cry1A.105* and 35S promoter regions did not show any significant differences for the cytosines residues analysed using Kruskal–Wallis test (*p* > 0.05) (Fig. 2c, d).

**Fig. 3:**
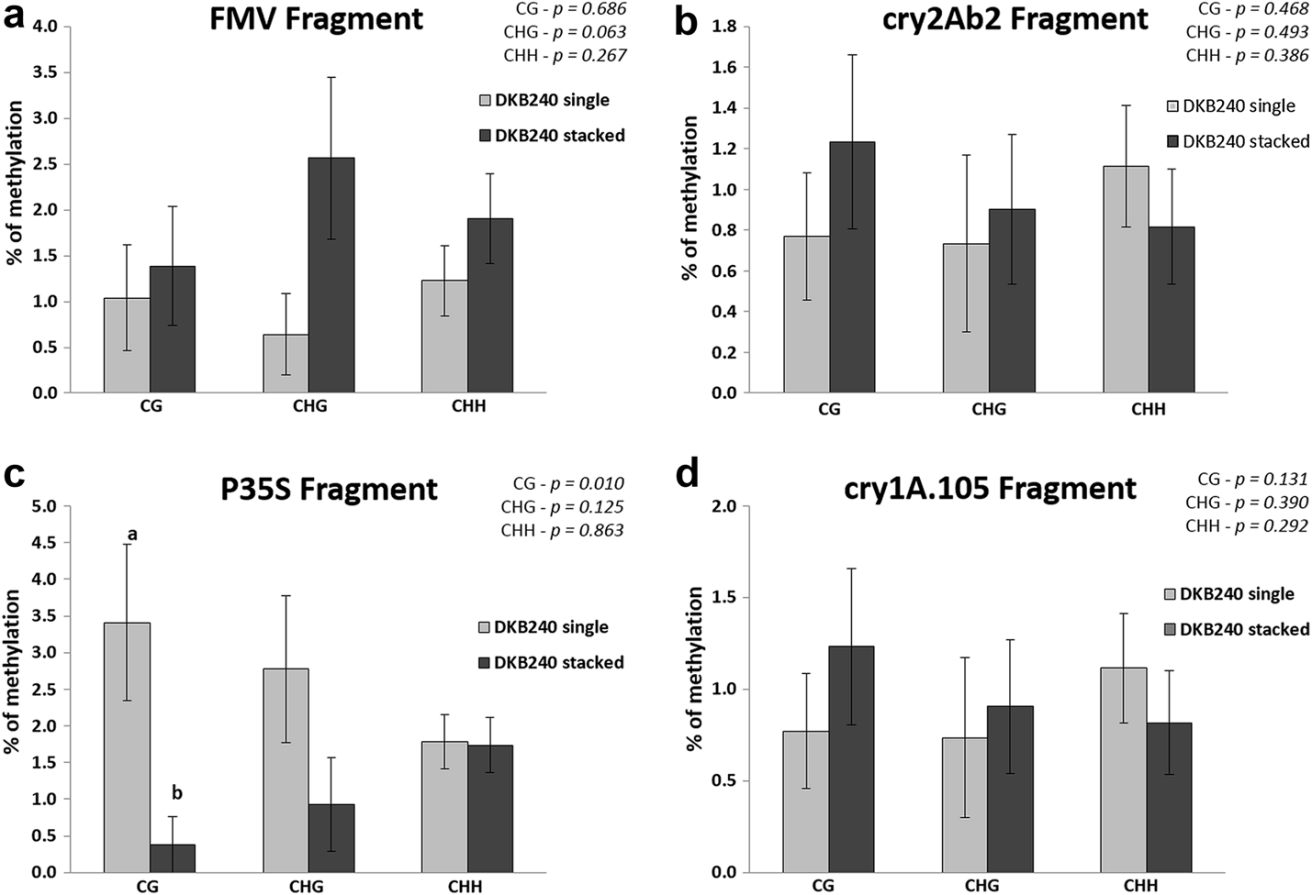
Cytosine methylation levels of single and stacked maize varieties in four regions of the transgenes. The levels of cytosine methylation were measured in CG, CHG and CHH residues. **a** FMV promoter region; **b** *cry2Ab2* transgene region; **c** 35S promoter region; **d** *cry1A.105* transgene region. *Vertical bars* indicate standard errors. Means followed by *different letters* in the same cytosine type are significantly different according to the Wilcoxon-Mann–Whitney test (*p* < 0.05). Exact *p* values for the statistical test of each of the cytosine comparisons are shown on the *right upper* corner

The second experimental setup compared the methylation pattern of transgenic regions on single and stacked transgenic events in the same genetic background (DKB240). The analysis was performed by the comparison of the DKB240 single GM maize (MON-89Ø34-3) with the DKB240 stacked GM maize (MON-89Ø34-3 × MON-ØØ6Ø3-6). Significant differences were found in the CG type for the 35S promoter region according to the Wilcoxon-Mann–Whitney test for non-parametric data (*p* = 0.010). The single event showed 3.40% of methylated cytosines, while the stacked showed 0.38% (Fig. 3c). Furthermore, the *cry1A.105* transgene, FMV promoter and *cry2Ab2* transgene regions did not show significant differences for the methylation analysis (Fig. 3a, b, d). The distribution of methylated and unmethylated cytosines for all samples in all the regions is shown in the Additional files 3, 4, 5 and 6.

### Transgene transcript accumulation in Bt-expressing maize varieties

We have further analysed the levels of transgene mRNA accumulation in the leaves of single and stacked maize varieties containing the *cry1A.105* and *cry2Ab2* transgenes by RT-qPCR in order to investigate if different methylation levels would result in differential mRNA accumulation. The DKB240 single GM maize was used as a reference sample for the transcript relative quantification analysis.

**Fig. 4:**
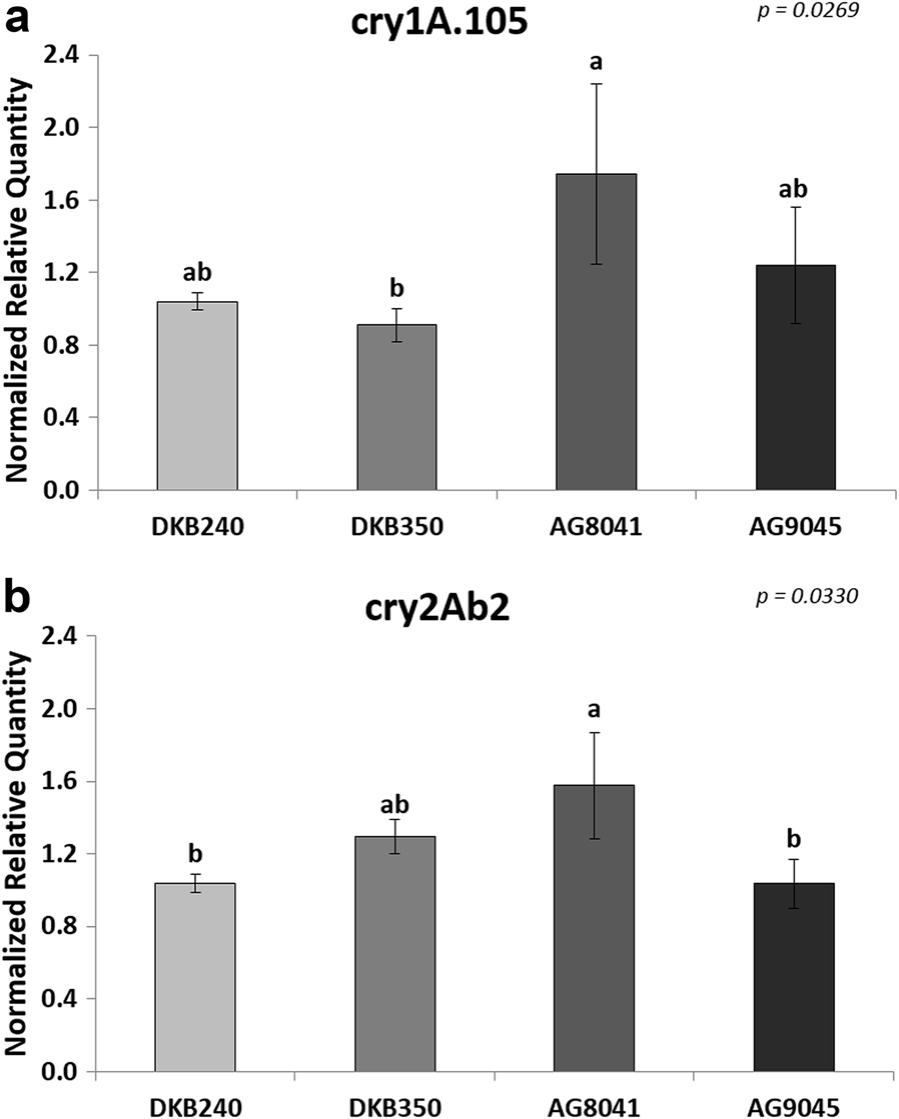
mRNA accumulation levels of transgenic transcripts among MON-89Ø34-3 maize varieties. **a** *cry1A.105* transgene; **b** *cry2Ab2* transgene. *Vertical bars* indicate standard deviation. *Different letters* above the *bars* indicate statistical differences among the samples at *p* < 0.05. Exact *p* value for the statistical test is shown on the *right upper* corner

The first experimental setup analysed the transgene transcript accumulation among four different single GM varieties. For the *cry1A.105* transcript analysis, AG8041 and AG9045 varieties showed a higher transcript accumulation (1.67- and 1.19-fold change, respectively), while the DKB350 variety showed a lower transcript accumulation (0.87-fold change). Statistically, AG8041 and DKB350 significantly differ between themselves, while DKB240 and AG9045 did not differ from any of the other varieties (Fig. 4a). For the accumulation of *cry2Ab2* transcripts, compared to the DKB240 variety, AG8041 and DKB350 varieties showed higher transcript accumulation levels (1.52- and 1.24-fold change, respectively), while the AG9045 showed a behaviour similar to the reference (0.99-fold change). Statistically, AG8041 significantly differ from DKB240 and AG9045, while DKB350 did not differ from any of the other varieties (Fig. 4b).

**Fig. 5:**
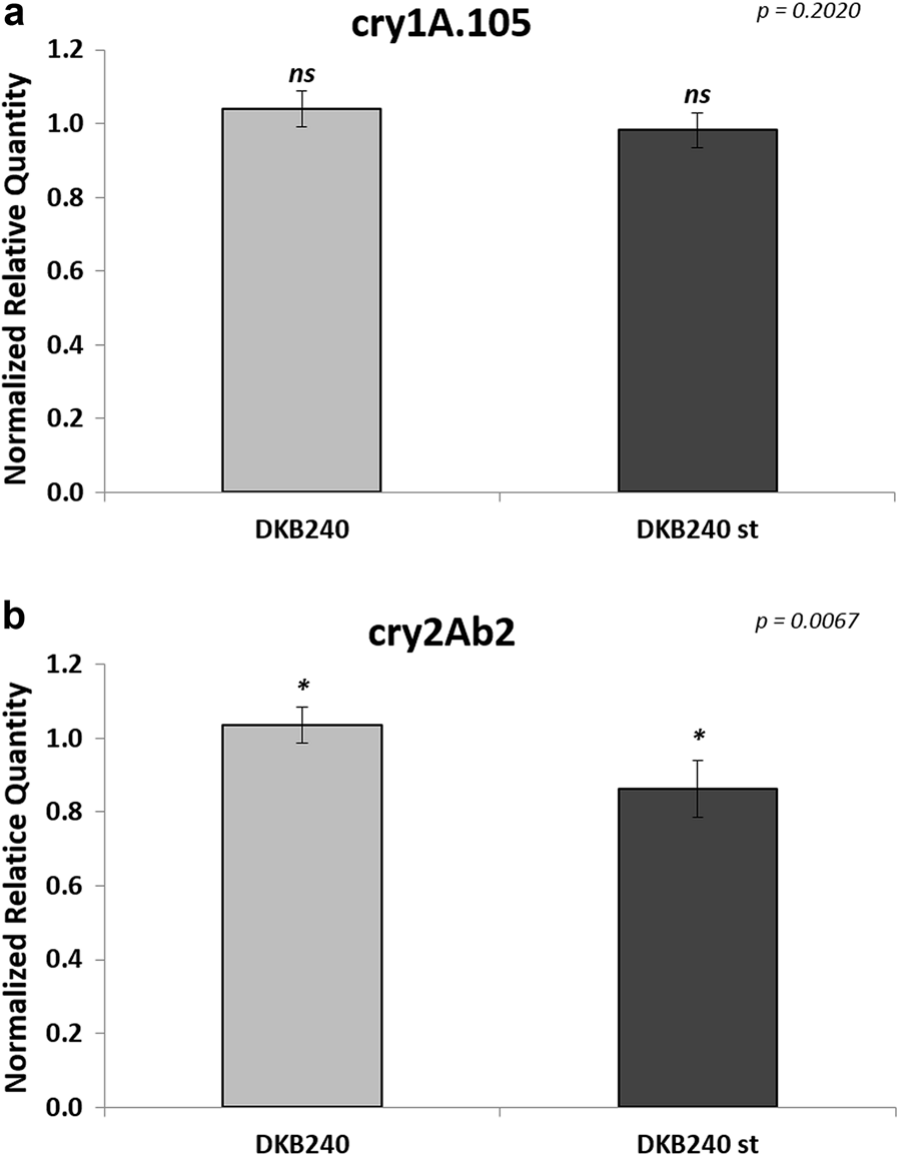
mRNA accumulation levels of transgenic transcripts between single and stacked maize varieties. **a** *cry1A.105* transgene; **b** *cry2Ab2* transgene. *Vertical bars* indicate standard deviation. *Asterisk* above the *bar* indicates statistical difference between the samples at *p* < 0.05. ‘*ns*’ above the *bar* indicates non-significant statistical difference (*p* > 0.05) between samples. Exact *p* value for the statistical test is shown on the *right upper* corner

The second experimental setup analysed the transgene transcript accumulation between a single GM variety and a stacked GM variety. The stacked event showed lower accumulation levels for the *cry1A.105* transgene (0.94-fold change), as well as for the *cry2Ab2* transgene (0.83-fold change) (Fig. 5a, b respectively). However, only the *cry2Ab2* transgene showed statistically significant differences (*p* = 0.0067).

## Discussion

### Cytosine methylation and its correlation with transgene transcript accumulation

When a transgenic event is approved for commercial use, it is required that the transgenic trait, or locus, is introgressed into local varieties. In such cases, the same transgenic event can be introgressed by traditional backcrosses into as many maize varieties as it is desirable. It is then expected that the transgenic insert is stable in all varieties (i.e. regarding its protein and transcript expression), in all available genetic background.

Epigenetic phenomena, such as DNA methylation, are known to affect transgene expression [47–49]. La Paz et al. [7] conducted the only study regarding epigenetic aspects of commercialized GM crops, in which they assessed the cytosine methylation levels of seven single GM maize varieties containing the MON810 transgenic event construct and did not find statistical differences in the 35S promoter and *cry1Ab* transgene regions. In contrast, our results show statistical differences between GM varieties in three of the four transgenic regions analysed, including the 35S promoter (when comparing single vs stacked varieties). However, La Paz et al. [7] results might have been hampered by a limited statistical power (caused by small sampling size), in which they analysed only one plant per variety and five bacteria colonies per plant, while our study analysed three plants per variety and eight bacteria colonies.

On the other hand, our results indeed showed high standard deviation values in the methylation analysis for all regions, which is explained by the high variation observed among the 8 selected clones. This variation might have impacted the significance of statistical analysis. Although bisulfite sequencing of single clones has been regarded as the gold standard of DNA methylation analysis for the past few years [50], there is no consensus of how many clones are adequate to provide reliable results. Distinct studies have used five [7, 51], six [52, 53] or ten clones [54, 55]; other studies do not even mention it within their “Methods” section [56, 57].

Transgene methylation has been long associated with TGS and PTGS, both in promoter [30] and coding sequence regions [58]. DNA methylation of promoter regions is generally associated with reduced expression of the regulated genes [59], whereas DNA methylation within gene bodies has a more complex association with gene expression, varying among species and level of gene expression [29, 59–61]. Although DNA methylation is not necessary for gene silencing [56], promoter methylation plays an important role in transgene silencing in vitro and in vivo [62]. Several studies have shown a correlation between gene expression and methylation levels of either promoter or gene regions [51, 52, 57, 63, 64].

Regarding the comparison between single and stacked GM varieties in our studies, the correlation between 35S promoter and *cry1A.105* transgene methylation and *cry1A.105* transcript accumulation was not robust, unlike the results in the previously mentioned studies. However, we did verify a correlation for the *FMV:cry2Ab2* transgene, where both FMV promoter and *cry2Ab2* sequences showed higher levels of CG and CHG methylation in the stacked event accompanied by a lower expression of the *cry2Ab2* transgene. The FMV (Figwort Mosaic Virus) is a double-stranded DNA virus with a genome of 8 kilobase pairs [65] and a genetic organization similar to that of cauliflower mosaic virus (CaMV) [66]. Therefore, due to the high similarity of those promoters, it is expected that the FMV promoter behaves similarly to the 35S, thus acting in the regulation of transgene expression.

### Transcript accumulation in Bt-expressing maize varieties

Our results show variation in the transgene transcript accumulation among single GM varieties, which was assessed by RT-qPCR analysis. One of the few studies performed with single GM maize has analysed *cry1Ab* mRNA accumulation in two MON810 varieties during several plant development stages, with the results showing certain levels of variation, but in the range of the natural variation [7]. Environmental conditions such as temperature and water accessibility also play an important role in transgene expression rate. This is further complicated by different genetic backgrounds in different GM varieties (even with the same event), which respond differently to the same environmental change with regards to transgenic mRNA expression [67].

Wang et al. [68] observed a variation in the levels of CRY1AB protein, measured by ELISA, among different cultivars of GM cotton. Luo et al. [69] submitted two Bt cotton cultivars, expressing the CRY1AC protein, under salinity stress. The levels of CRY1*A* protein, measured by ELISA, varied between cultivars, with one of the cultivars showing lower CRY1A content earlier than the other in comparison to the controls. Other studies with Bt cotton have also reported variation of transgenic protein among cultivars containing the same GM construct [70, 71], which indicates that genetic background might influence the levels of transgene expression.

Nevertheless, changes in transgene expression levels due to differences in genetic background may have an impact in the safety and utility of GM plants. Koul et al. [72] showed a correlation between transcript accumulation and Bt protein in different transgenic tomato lines expressing the *cry1Ab* transgene. Olsen et al. [73] found that the developmental decline in bioefficacy (against *Helicoverpa armigera*) in field-grown plants was associated with reduced *cry1Ac* transcript levels and Bt toxin levels in postsquaring cotton. The authors were also able to corroborate the relationship between the decline in efficacy and reduced mRNA levels with plants grown in contention. The biggest safety concern with the variation of transgenic transcript and protein accumulation is related to the possibility of field-evolved resistance. Cases of resistance of target organism to Bt toxins have been report [74, 75], and they were usually related to a low adherence of farmers in cultivating a refuge area with non-GM varieties [76]. However, variation on the toxin doses can also be linked as one of the causes for the appearance of resistant insects [77, 78]. The variation of Bt toxins has been previously linked to environmental conditions and genetic background [79, 80], but the reason remains unclear and might be linked to mRNA instability, variation in promoter activity, reduction in protein expression and protein–protein interactions [42, 73].

Variation on the levels of transgene expression is widely known, but its cause has not been fully understood yet [81]. An active transgenes might be silenced through the introduction in its genome of a second transgene regulated by the same promoter [82]. In addition, there are reports showing that gene silencing is frequently associated with multiple transgene integrations due to homology between the transgenes [83–85]. In fact, DNA homology has long been suggested as a mechanism for two trans-inactivation systems comprising a silencing transgene locus and a target transgene locus that share homology only in promoter regions [86, 87]. In the NOS promoter-based system, silencing phenomena was observed with a promoter homology of about 300 bp [88]. In the 35S promoter-based system, however, silencing was observed even when promoter homology comprised only 90 bp [86]. Even though 90 bp seems a short sequence for direct DNA–DNA pairing, it still does not preclude the recognition of DNA homology [89]. Mishiba et al. [52] report that gentian plants showed no expression of *bar* and *GtMADS* genes, under the regulation of the 35S promoter, and that the possible cause for the silencing phenomenon might be homology-dependent gene silencing involving the promoter regions.

Agapito-Tenfen et al. [42] also found reduced transgene expression in stacked transgenes with the same GM event, but with different varieties. The authors suggested that these reductions might be related to the high energetic demand of the cell. In this aspect, evidences support the idea that constitutive promoters involve a high energetic cost, leading to a penalty in transgenic plants [90–92]. Moreover, the authors have also performed a proteomic profiling of single and stacked GM, and some metabolic pathways, such as energy/carbohydrate metabolism and detoxification metabolism pathways, were differentially modulated. These results suggest that the insertion of a new transgene in a single GM plant may alter the expression of endogenous genes, especially those related to energetic metabolism, since the transgene are being expressed constantly in the plant tissues and, therefore, demand high levels of energy.

Even though a quantification of transgenic proteins was not performed in our study, the transgene analysis (in our case *cry1A.105* and *cry2Ab2* transgenes) of transcripts accumulation showed some variation among varieties. Therefore, additional studies could be performed in order to further investigate the correlation of transgene transcripts and levels of Bt toxin production, and even the bioefficacy of the transgenic proteins against the target organisms, and thus the biological meaning behind it. Taken together, our results are in agreement to that of our previous work that showed reduced transgene expression in stacked transgenes with the same GM event, but with different varieties [42]. We have observed a significant reduction of the expression of the *cry2Ab2* transgenes in the stacked event. Safety implications are related to cases in which the expression level of an introduced/modified trait in a GM stacked event falls outside the range of what was determined in the parental line, and a re-evaluation of the environmental aspects might be necessary [93].

### Contributions to risk assessment of GM crops

Regulatory practice within the European Union (EU) consider stacked events as new GM organisms, and additional information on the stability of transgene insertions, expression levels and potential antagonistic or synergistic interactions should be provided prior to marketing [93–95]. Overall, there is a few available data on transgene expression levels in both stacked and single transgene GM crops in the scientific literature. Although data on expression levels for stacked GM events are required for approval according to EU regulations (No 503/2013), these are rarely disclosed or they are considered insufficient [42, 96, 97]. Recent discussions about potential risks of stacked events, as well as the opinion of the European Food Safety Authority (EFSA) on those issues, have highlighted the lack of consensus with regard to whether such GMOs should be subject to specific assessments [98].

As for the DNA methylation data, there is also a lack of scientific literature for any GM crop, and the regulations do not consider yet the need for this type of analysis. Data related to possible epigenetic variations of GM crops, such as DNA methylation levels and ncRNAs, could help to extend our knowledge on the safety of such organism and, therefore, may be taken in account in risk assessments. While there is a lack of specific investigation of cytosine methylation levels in commercialized transgenic crops, the literature records show that methylation of promoter regions and coding sequences may also result in decreased levels of transgene transcription [51, 57, 63]. The same can be applied to ncRNAs, where studies have reported the existence of gene silencing mediated by 35S promoter homology between transgenes; however, whether the silencing is mediated by 35S promoter siRNAs produced from complex transgene inserts is still not fully known [99]. Moreover, the 35S promoter can also be silenced and methylated by the production of homologous siRNAs [100, 101]. Since both *epsps* and *cry1A.105* transgenes present in the stacked line used in this study are controlled by homologous 35S promoters, TGS, PTGS or other processes being involved in transgene transcript modulation in the stacked line cannot be ruled out.

So far, no other study has compared the methylation levels of transgene sequences of this particular GM event in both single and stacked varieties, and its possible correlation with transgene transcript accumulation. It is important to emphasize that the findings of the present study are restricted to the set of varieties used. We cannot extrapolate this information to other single or stacked GM varieties without testing it. Hence, there is a lack of this kind of data that might be important in order to reliably assess the safety of stacked and single GM events.

## Conclusions

In conclusion, our results showed that DNA methylation levels of transgenic sequences vary among single GM maize varieties and between single and stacked GM maize varieties. We also observed that the accumulation of transgene transcript showed variation among single GM varieties, which indicates that genetic background might have some influence in the levels of transgene expression. Likewise, accumulation of transgene transcript also varied between single and stacked GM varieties, with the stacked one showing a statistically significant reduction for the *cry2Ab2* transgene.

These conclusions arose from the statistically different levels of DNA methylation in the FMV promoter and *cry2Ab2* gene among single GM varieties and in the 35S promoter for the comparison of single and stacked GM varieties. In addition, transgenic transcript accumulation levels demonstrate a high variation between single GM varieties, with samples showing an increase up to 1.67-fold change and a decrease of 0.87-fold change in accumulation compared to the reference variety (for the *cry1A.105* transgene). Moreover, transgenic transcript accumulation levels in the stacked GM variety showed a reduction of about 0.94- and 0.87-fold change (*cry1A.105* and *cry2Ab2* transgenes, respectively) when compared to parental single event.

Similar results of transgenic transcript accumulation have been reported in our previous study. However, this is one of the first studies that assessed and verified changes in methylation levels of transgenic sequences of single and stacked GM crops. Although the detection of changes in DNA methylation levels and transgenic accumulation levels does not present a safety issue per se, it demonstrates the need for additional studies that address the biological relevance and the possible safety implications of such changes.

## Additional files

**Additional file 1.** Output results of NormFinder algorithm used for determining the best endogenous normalizer genes. *Leunig* (LUG) and *Membrane protein PB1A10.07c* **(**MEP) showed the best combination of two normalizer genes with a stability value of 0.025.

**Additional file 2.** Alignment of the 35S promoters from the *CP4-epsps* (Query) and *cry1A.105* (Sbjct). The sequence in bold and underlined corresponds to the region where the BS35S-F primer was designed.

**Additional file 3.** Position and distribution of methylated and unmethylated cytosines for the 35S promoter sequence in all analysed hybrids. Each line corresponds to one sequenced bacteria colony (clone). Clones from the same hybrid contain the same nomenclature, ranging from 1-24. Class I: CG residue; Class II: CHG residue; Class III: CHH residue. Filled icons: methylated cytosine; Empty icons: non-methylated cytosines.

**Additional file 4.** Position and distribution of methylated and unmethylated cytosines for the *cry1A.105* gene sequence in all analysed hybrids. Each line corresponds to one sequenced bacteria colony (clone). Clones from the same hybrid contain the same nomenclature, ranging from 1–24. Class I: CG residue; Class II: CHG residue; Class III: CHH residue. Filled icons: methylated cytosine; Empty icons: non-methylated cytosines.

**Additional file 5.** Position and distribution of methylated and unmethylated cytosines for the FMV promoter sequence in all analysed hybrids. Each line corresponds to one sequenced bacteria colony (clone). Clones from the same hybrid contain the same nomenclature, ranging from 1–24. Class I: CG residue; Class II: CHG residue; Class III: CHH residue. Filled icons: methylated cytosine; Empty icons: non-methylated cytosines.

**Additional file 6.** Position and distribution of methylated and unmethylated cytosines for the *cry2Ab2* gene sequence in all analysed hybrids. Each line corresponds to one sequenced bacteria colony (clone). Clones from the same hybrid contain the same nomenclature, ranging from 1–24. Class I: CG residue; Class II: CHG residue; Class III: CHH residue. Filled icons: methylated cytosine; Empty icons: non-methylated cytosines.
