# Supplementary figures and images for: Levels of DNA methylation and transcript accumulation in leaves of transgenic maize varieties
Source: Environ Sci Eur. 2016 Nov 23;28(1):29. doi: 10.1186/s12302-016-0097-2 (PMC5120055; doi:10.1186/s12302-016-0097-2)

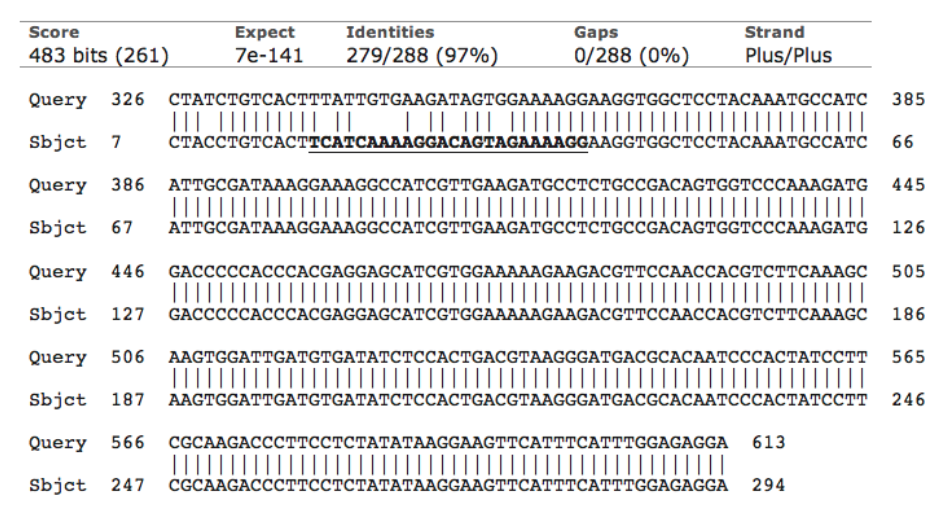

Supplement: Supplementary file 2 — Additional file 2. Alignment of the 35S promoters from the CP4-epsps (Query) and cry1A.105 (Sbjct). The sequence in bold and underlined corresponds to the region where the BS35S-F primer was designed. [file 12302_2016_97_MOESM2_ESM.tiff]
