# Supplementary material for: Levels of DNA methylation and transcript accumulation in leaves of transgenic maize varieties
Source: Environ Sci Eur. 2016 Nov 23;28(1):29. doi: 10.1186/s12302-016-0097-2 (PMC5120055; doi:10.1186/s12302-016-0097-2)
Supplement: Supplementary file 3 — Additional file 3. Position and distribution of methylated and unmethylated cytosines for the 35S promoter sequence in all analysed hybrids. Each line corresponds to one sequenced bacteria colony (clone). Clones from the same hybrid contain the same nomenclature, ranging from 1-24. Class I: CG residue; Class II: CHG residue; Class III: CHH residue. Filled icons: methylated cytosine; Empty icons: non-methylated cytosines. [file 12302_2016_97_MOESM3_ESM.pdf]

CyMATE (c) 2007, 2008

Methylation overview of '35S_fragment.afa'

Class 1: ● me ○ not me
Class 2: ■ me □ not me
Class 3: ▶ me ▷ not me

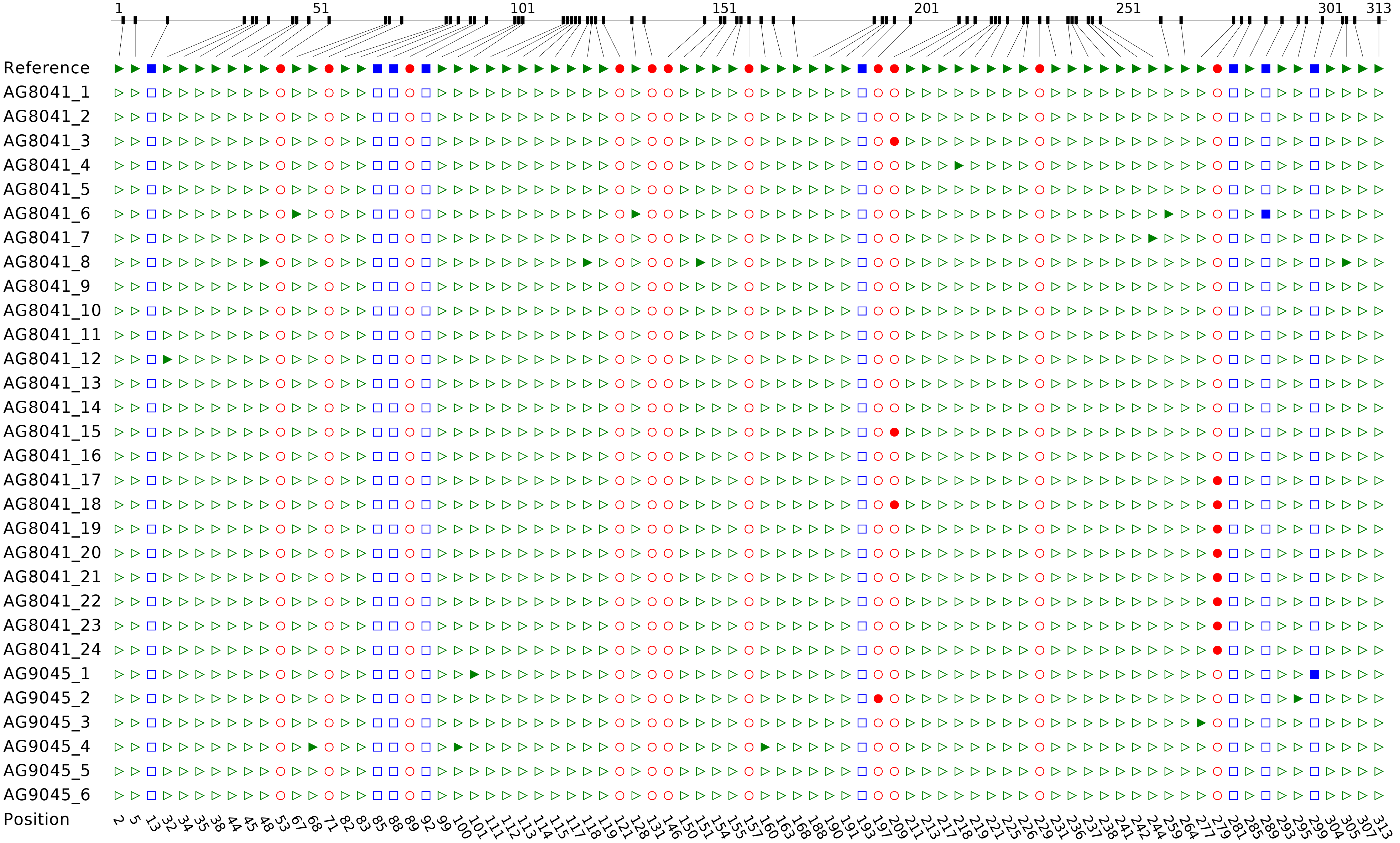

CyMATE (c) 2007, 2008

Methylation overview of '35S_fragment.afa'

Class 1:	● me	○ not me
Class 2:	■ me	□ not me
Class 3:	▶ me	▷ not me

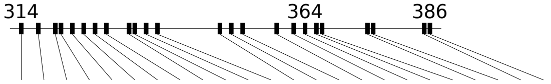

Position	314	317	320	321	323	325	327	329	333	334	336	338	349	351	353	359	362	364	366	367	375	376	385	386
Reference	▶	▶	▶	▶	▶	▶	▶	▶	▶	▶	▶	▶	▶	▶	■	▶	▶	▶	▶	▶	▶	▶	▶	▶
AG8041_1	▷	▷	▷	▷	▷	▷	▷	▷	▷	▷	▷	▷	▷	▷	□	▷	▷	▷	▷	▷	▷	▷	▷	▷
AG8041_2	▷	▷	▷	▷	▷	▷	▷	▷	▷	▷	▷	▷	▷	▷	□	▷	▷	▷	▷	▷	▷	▷	▷	▷
AG8041_3	▷	▷	▷	▷	▷	▷	▷	▷	▷	▷	▷	▷	▷	▷	□	▷	▷	▷	▷	▷	▷	▷	▷	▷
AG8041_4	▷	▷	▷	▷	▷	▷	▷	▷	▷	▷	▷	▷	▷	▶	□	▷	▷	▷	▷	▷	▷	▷	▷	▷
AG8041_5	▷	▷	▷	▷	▷	▷	▷	▷	▷	▶	▷	▷	▷	▷	□	▷	▷	▷	▷	▷	▷	▷	▷	▷
AG8041_6	▷	▷	▷	▷	▷	▷	▷	▷	▷	▷	▷	▶	▷	▷	□	▷	▷	▷	▷	▷	▷	▷	▷	▷
AG8041_7	▷	▷	▷	▷	▷	▷	▷	▷	▷	▶	▷	▷	▷	▷	□	▷	▷	▷	▷	▷	▷	▷	▷	▷
AG8041_8	▷	▷	▷	▷	▷	▷	▷	▷	▷	▷	▷	▷	▷	▷	□	▷	▷	▷	▷	▷	▷	▷	▷	▷
AG8041_9	▷	▷	▷	▷	▷	▷	▷	▷	▷	▷	▷	▷	▷	▷	□	▷	▷	▷	▷	▷	▷	▷	▷	▷
AG8041_10	▷	▷	▷	▷	▷	▷	▷	▷	▷	▷	▷	▷	▷	▷	□	▷	▷	▷	▷	▷	▷	▷	▷	▷
AG8041_11	▷	▷	▷	▷	▷	▷	▷	▷	▷	▷	▷	▷	▷	▷	□	▷	▷	▷	▷	▷	▷	▷	▷	▷
AG8041_12	▷	▷	▷	▷	▷	▷	▷	▷	▷	▷	▷	▷	▷	▷	□	▷	▷	▷	▷	▷	▷	▷	▷	▷
AG8041_13	▷	▷	▷	▷	▷	▷	▷	▷	▷	▷	▷	▷	▷	▷	□	▷	▷	▷	▷	▷	▷	▷	▷	▷
AG8041_14	▷	▷	▷	▷	▷	▷	▷	▷	▷	▷	▷	▷	▷	▷	□	▷	▷	▷	▷	▷	▷	▷	▷	▷
AG8041_15	▷	▷	▷	▷	▷	▷	▷	▷	▷	▷	▷	▷	▷	▷	□	▷	▷	▷	▷	▷	▷	▷	▷	▷
AG8041_16	▷	▷	▷	▷	▷	▷	▷	▷	▷	▷	▷	▷	▷	▷	□	▷	▷	▷	▷	▷	▷	▷	▷	▷
AG8041_17	▷	▷	▷	▷	▷	▷	▷	▷	▷	▷	▷	▷	▷	▷	□	▷	▷	▶	▷	▷	▷	▷	▷	▷
AG8041_18	▷	▷	▷	▷	▷	▷	▷	▷	▷	▷	▷	▷	▷	▷	□	▷	▷	▶	▷	▷	▷	▷	▷	▷
AG8041_19	▷	▷	▷	▷	▷	▷	▷	▷	▷	▷	▷	▷	▷	▷	□	▷	▷	▶	▷	▷	▷	▷	▷	▷
AG8041_20	▷	▷	▷	▷	▷	▷	▷	▷	▷	▷	▷	▷	▷	▷	□	▷	▷	▶	▷	▷	▷	▷	▷	▷
AG8041_21	▷	▷	▷	▷	▷	▷	▷	▷	▷	▷	▷	▷	▷	▷	□	▷	▷	▶	▷	▷	▷	▷	▷	▷
AG8041_22	▷	▷	▷	▷	▷	▷	▷	▷	▷	▷	▷	▷	▷	▷	□	▷	▷	▶	▷	▷	▷	▷	▷	▷
AG8041_23	▷	▷	▷	▷	▷	▷	▷	▷	▷	▷	▷	▷	▷	▷	□	▷	▷	▶	▷	▷	▷	▷	▷	▷
AG8041_24	▷	▷	▷	▷	▷	▷	▷	▷	▷	▷	▷	▷	▷	▷	□	▷	▷	▶	▷	▷	▷	▷	▷	▷
AG9045_1	▷	▷	▷	▷	▷	▶	▷	▷	▷	▷	▷	▷	▷	▷	□	▷	▷	▷	▷	▷	▷	▷	▷	▷
AG9045_2	▷	▷	▷	▷	▷	▷	▷	▷	▷	▷	▷	▷	▷	▷	□	▷	▷	▷	▷	▷	▷	▷	▷	▷
AG9045_3	▷	▷	▷	▷	▷	▷	▷	▷	▷	▷	▷	▷	▷	▷	□	▷	▷	▷	▷	▷	▷	▷	▷	▷
AG9045_4	▷	▶	▷	▷	▷	▷	▷	▷	▷	▷	▷	▷	▷	▶	□	▷	▷	▷	▷	▷	▷	▷	▷	▷
AG9045_5	▷	▷	▷	▷	▷	▷	▷	▷	▷	▷	▷	▷	▷	▷	□	▷	▷	▷	▷	▷	▷	▷	▷	▷
AG9045_6	▷	▷	▷	▷	▷	▷	▷	▷	▷	▷	▷	▷	▷	▷	□	▷	▷	▷	▷	▷	▷	▷	▷	▷

CyMATE (c) 2007, 2008

Methylation overview of '35S_fragment.afa'

Class 1: ● me ○ not me

Class 2: ■ me □ not me

Class 3: ▶ me ▷ not me

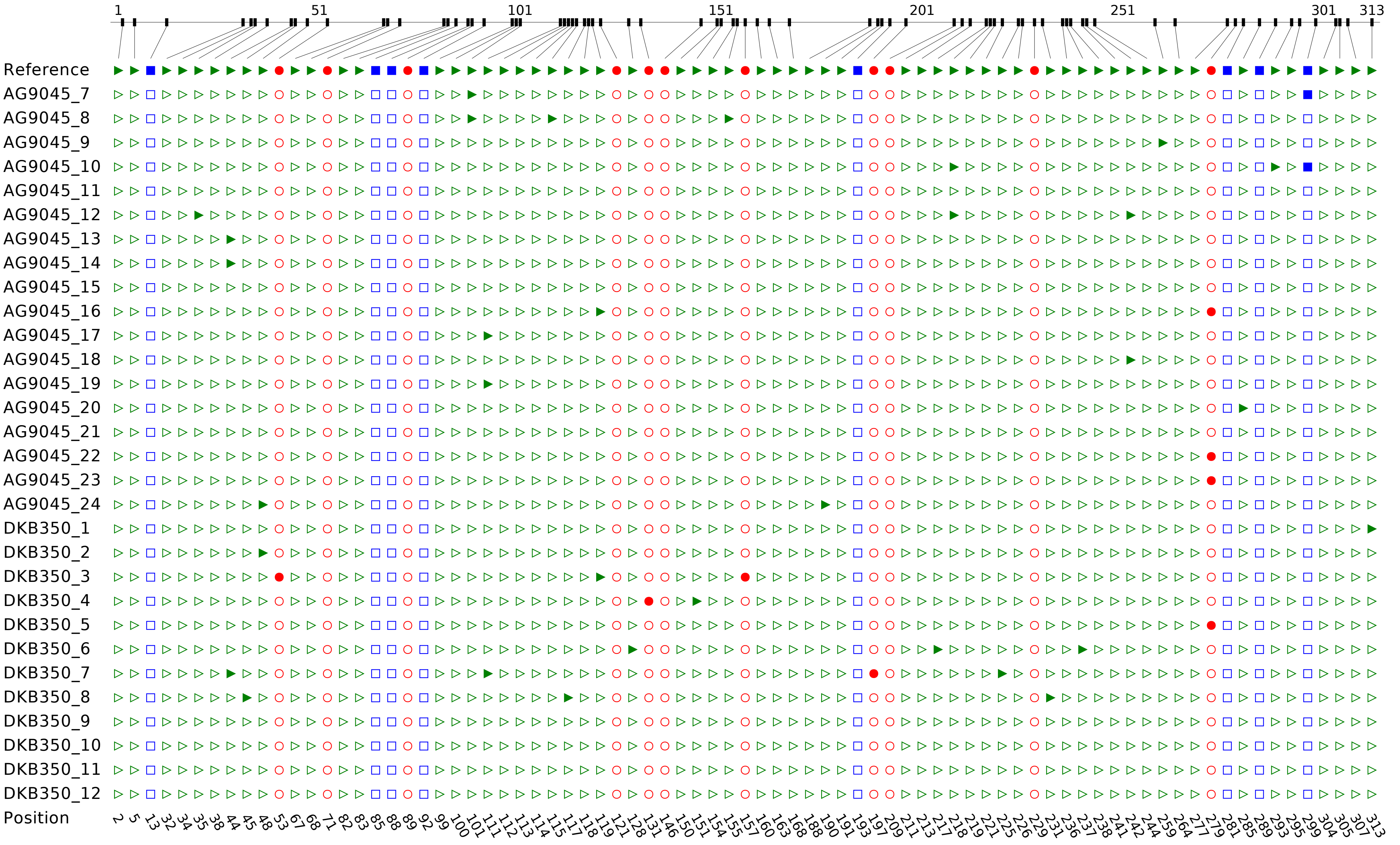

CyMATE (c) 2007, 2008

Methylation overview of '35S_fragment.afa'

Class	me	not me
Class 1:	● me	○ not me
Class 2:	■ me	□ not me
Class 3:	▶ me	▷ not me

314 364 386

Position	314	317	320	321	323	325	327	329	333	334	336	338	349	351	353	359	362	364	366	367	375	376	385	386
Reference	▶	▶	▶	▶	▶	▶	▶	▶	▶	▶	▶	▶	▶	▶	■	▶	▶	▶	▶	▶	▶	▶	▶	▶
AG9045_7	▷	▷	▷	▷	▷	▶	▷	▷	▷	▷	▷	▷	▷	▷	□	▷	▷	▷	▷	▷	▷	▷	▷	▷
AG9045_8	▷	▷	▷	▷	▷	▷	▷	▷	▷	▷	▷	▷	▷	▶	□	▷	▷	▷	▷	▷	▷	▷	▷	▷
AG9045_9	▷	▷	▷	▷	▷	▷	▷	▷	▷	▷	▷	▷	▷	▷	□	▷	▷	▷	▷	▷	▷	▷	▷	▷
AG9045_10	▷	▷	▷	▷	▶	▷	▷	▷	▷	▷	▷	▷	▷	▷	□	▷	▷	▷	▷	▷	▷	▷	▷	▷
AG9045_11	▷	▷	▷	▷	▷	▷	▷	▷	▷	▷	▷	▷	▷	▷	□	▷	▷	▷	▷	▷	▷	▷	▷	▷
AG9045_12	▷	▷	▷	▷	▷	▷	▷	▷	▷	▷	▷	▷	▷	▷	□	▷	▷	▷	▷	▷	▷	▷	▷	▷
AG9045_13	▷	▷	▷	▷	▷	▷	▷	▷	▷	▶	▷	▷	▷	▷	□	▷	▷	▷	▷	▷	▷	▷	▷	▷
AG9045_14	▷	▷	▷	▷	▷	▷	▷	▷	▷	▶	▷	▷	▷	▷	□	▷	▷	▷	▷	▷	▷	▷	▷	▷
AG9045_15	▷	▷	▷	▷	▷	▷	▷	▷	▷	▷	▷	▷	▷	▷	□	▷	▷	▷	▷	▷	▷	▷	▷	▷
AG9045_16	▷	▷	▷	▷	▷	▷	▷	▷	▷	▷	▷	▷	▷	▷	□	▷	▷	▷	▷	▷	▷	▷	▷	▷
AG9045_17	▷	▷	▷	▷	▷	▷	▷	▷	▷	▷	▷	▷	▷	▷	□	▷	▷	▷	▷	▷	▷	▷	▷	▷
AG9045_18	▷	▷	▷	▷	▷	▷	▷	▷	▷	▷	▷	▷	▷	▷	□	▷	▷	▷	▷	▷	▷	▷	▷	▷
AG9045_19	▷	▷	▷	▷	▷	▷	▷	▷	▷	▷	▷	▷	▷	▷	□	▷	▷	▷	▷	▷	▷	▷	▷	▷
AG9045_20	▷	▷	▷	▷	▷	▷	▷	▷	▷	▷	▷	▷	▷	▷	□	▷	▷	▷	▷	▷	▷	▷	▷	▷
AG9045_21	▷	▷	▷	▷	▷	▷	▷	▷	▷	▷	▷	▷	▷	▷	□	▷	▷	▷	▷	▷	▷	▷	▷	▷
AG9045_22	▷	▷	▷	▷	▷	▷	▷	▷	▷	▷	▷	▷	▷	▷	□	▷	▷	▶	▷	▷	▷	▷	▷	▷
AG9045_23	▷	▷	▷	▷	▷	▷	▷	▷	▷	▷	▷	▷	▷	▷	□	▷	▷	▶	▷	▷	▷	▷	▷	▷
AG9045_24	▷	▷	▷	▷	▷	▷	▷	▷	▷	▷	▷	▷	▷	▶	□	▷	▷	▷	▷	▷	▷	▷	▷	▷
DKB350_1	▷	▷	▷	▷	▷	▷	▷	▷	▷	▷	▷	▷	▷	▷	□	▷	▷	▷	▷	▷	▷	▷	▷	▷
DKB350_2	▷	▷	▷	▷	▷	▷	▷	▷	▷	▷	▶	▷	▷	▷	□	▷	▷	▷	▷	▷	▷	▷	▷	▷
DKB350_3	▷	▷	▷	▶	▷	▷	▷	▷	▷	▷	▶	▷	▷	▷	□	▷	▷	▶	▷	▷	▷	▷	▷	▷
DKB350_4	▷	▷	▷	▷	▷	▷	▷	▷	▷	▷	▷	▷	▷	▷	□	▷	▷	▷	▷	▷	▷	▷	▷	▷
DKB350_5	▷	▷	▷	▷	▷	▷	▷	▷	▷	▷	▷	▷	▷	▷	□	▷	▷	▶	▷	▷	▷	▷	▷	▷
DKB350_6	▷	▷	▷	▷	▷	▷	▷	▷	▷	▷	▷	▷	▷	▷	□	▷	▷	▷	▷	▷	▷	▷	▷	▷
DKB350_7	▷	▷	▷	▷	▶	▷	▷	▷	▷	▷	▷	▷	▷	▶	□	▷	▷	▶	▷	▷	▷	▷	▷	▷
DKB350_8	▷	▷	▷	▷	▷	▷	▶	▷	▷	▷	▷	▷	▶	▷	□	▷	▷	▷	▷	▷	▷	▷	▷	▷
DKB350_9	▷	▷	▷	▷	▷	▷	▷	▷	▷	▷	▷	▷	▷	▷	□	▷	▷	▷	▷	▷	▷	▷	▷	▷
DKB350_10	▷	▷	▷	▷	▷	▷	▷	▷	▷	▷	▷	▷	▷	▷	□	▷	▷	▷	▷	▷	▷	▷	▷	▷
DKB350_11	▷	▷	▷	▷	▷	▷	▷	▷	▷	▷	▷	▷	▷	▷	□	▷	▷	▶	▷	▷	▷	▷	▷	▷
DKB350_12	▷	▷	▶	▷	▷	▷	▷	▷	▷	▷	▷	▷	▷	▷	□	▷	▷	▷	▷	▷	▷	▷	▷	▷

CyMATE (c) 2007, 2008
Methylation overview of '35S_fragment.afa'

Class 1: ● me ○ not me
Class 2: ■ me □ not me
Class 3: ▶ me ▷ not me

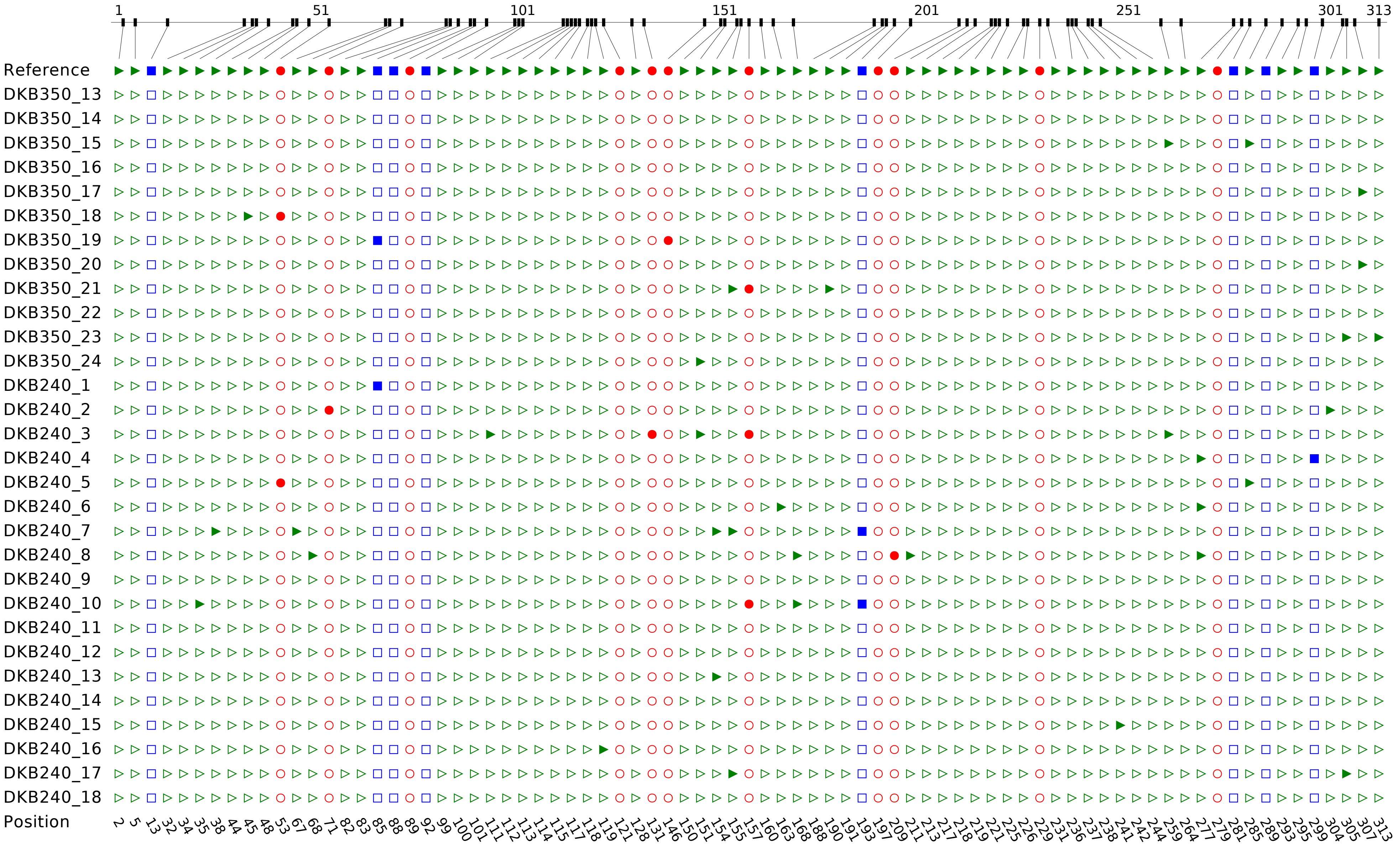

CyMATE (c) 2007, 2008

Methylation overview of '35S_fragment.afa'

Class 1: ● me ○ not me

Class 2: ■ me □ not me

Class 3: ▶ me ▷ not me

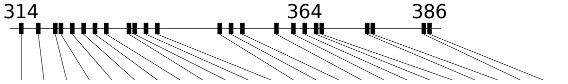

	314	317	320	321	323	325	327	329	333	334	336	338	349	351	353	359	362	364	366	367	375	376	385	386
Reference	▶	▶	▶	▶	▶	▶	▶	▶	▶	▶	▶	▶	▶	▶	■	▶	▶	▶	▶	▶	▶	▶	▶	▶
DKB350_13	▷	▷	▷	▷	▷	▷	▷	▷	▷	▷	▷	▷	▷	▷	□	▷	▷	▷	▷	▷	▷	▷	▷	▷
DKB350_14	▷	▷	▷	▷	▷	▷	▷	▷	▷	▷	▷	▷	▷	▷	□	▷	▷	▷	▷	▷	▷	▷	▷	▷
DKB350_15	▷	▷	▷	▷	▷	▷	▷	▷	▷	▷	▷	▷	▷	▷	□	▷	▷	▷	▷	▷	▷	▷	▷	▷
DKB350_16	▶	▷	▷	▷	▷	▷	▷	▷	▷	▷	▷	▷	▷	▶	□	▷	▷	▷	▷	▷	▷	▷	▷	▷
DKB350_17	▷	▷	▷	▷	▷	▷	▷	▷	▷	▷	▷	▷	▷	▷	□	▷	▷	▷	▷	▷	▷	▷	▷	▷
DKB350_18	▷	▷	▷	▷	▷	▷	▷	▷	▷	▷	▷	▷	▷	▷	□	▶	▷	▷	▷	▷	▷	▷	▷	▷
DKB350_19	▷	▷	▷	▷	▷	▷	▶	▷	▷	▷	▷	▷	▷	▷	□	▷	▷	▷	▷	▷	▷	▷	▷	▷
DKB350_20	▷	▷	▷	▷	▷	▷	▷	▷	▷	▷	▷	▷	▷	▷	□	▷	▷	▷	▷	▷	▷	▷	▷	▷
DKB350_21	▷	▷	▷	▷	▷	▷	▶	▷	▷	▶	▷	▷	▷	▷	□	▷	▶	▷	▷	▷	▷	▷	▷	▷
DKB350_22	▷	▷	▷	▷	▷	▷	▷	▷	▷	▷	▷	▷	▷	▷	□	▷	▷	▷	▷	▷	▷	▷	▷	▷
DKB350_23	▷	▷	▷	▷	▷	▷	▷	▷	▷	▷	▷	▷	▷	▷	□	▷	▷	▷	▷	▷	▷	▷	▷	▷
DKB350_24	▷	▷	▷	▷	▷	▷	▷	▷	▷	▷	▷	▷	▷	▷	□	▷	▷	▷	▷	▷	▷	▷	▷	▷
DKB240_1	▷	▷	▷	▷	▶	▷	▷	▷	▷	▷	▷	▷	▷	▷	□	▷	▷	▷	▷	▷	▷	▷	▷	▷
DKB240_2	▷	▷	▷	▷	▷	▷	▷	▷	▷	▷	▷	▷	▷	▷	□	▷	▷	▷	▷	▷	▷	▷	▷	▷
DKB240_3	▶	▷	▷	▷	▷	▷	▷	▷	▷	▷	▷	▷	▷	▷	□	▷	▷	▷	▷	▷	▷	▷	▷	▷
DKB240_4	▷	▷	▷	▷	▷	▷	▷	▷	▷	▷	▷	▷	▷	▷	□	▷	▷	▷	▷	▷	▷	▷	▷	▷
DKB240_5	▷	▷	▷	▷	▷	▷	▷	▷	▷	▷	▷	▷	▷	▷	□	▷	▷	▷	▷	▷	▷	▷	▷	▷
DKB240_6	▷	▷	▷	▷	▷	▷	▷	▷	▷	▷	▷	▷	▷	▷	□	▷	▷	▷	▷	▷	▷	▷	▷	▷
DKB240_7	▷	▷	▷	▷	▷	▷	▷	▷	▷	▷	▷	▷	▷	▷	□	▷	▷	▷	▷	▷	▷	▷	▷	▷
DKB240_8	▷	▷	▷	▶	▷	▶	▷	▷	▷	▷	▷	▷	▷	▷	□	▷	▷	▷	▷	▷	▷	▷	▷	▷
DKB240_9	▷	▷	▷	▷	▷	▷	▷	▷	▷	▷	▷	▶	▷	▷	□	▷	▷	▷	▷	▷	▷	▷	▷	▷
DKB240_10	▷	▷	▷	▷	▷	▷	▷	▷	▷	▷	▶	▷	▷	▷	□	▷	▷	▷	▷	▷	▷	▷	▷	▷
DKB240_11	▷	▷	▷	▷	▷	▷	▷	▷	▷	▷	▷	▷	▷	▷	□	▷	▷	▷	▷	▷	▷	▷	▷	▷
DKB240_12	▷	▷	▷	▷	▷	▷	▷	▷	▷	▷	▷	▷	▷	▷	□	▷	▷	▷	▷	▷	▷	▷	▷	▷
DKB240_13	▷	▷	▷	▷	▷	▷	▷	▷	▷	▷	▷	▷	▷	▷	□	▷	▷	▷	▷	▷	▷	▷	▷	▷
DKB240_14	▷	▷	▷	▷	▷	▷	▷	▷	▷	▷	▷	▷	▷	▷	□	▷	▷	▷	▷	▷	▷	▷	▷	▷
DKB240_15	▷	▷	▷	▷	▷	▷	▷	▷	▷	▷	▷	▷	▷	▷	□	▷	▷	▷	▷	▷	▷	▷	▷	▷
DKB240_16	▷	▷	▷	▷	▷	▷	▷	▷	▷	▷	▷	▷	▷	▷	□	▷	▷	▷	▷	▷	▷	▷	▷	▷
DKB240_17	▷	▷	▷	▷	▷	▷	▷	▷	▷	▷	▷	▷	▷	▷	□	▷	▷	▷	▷	▷	▷	▷	▷	▷
DKB240_18	▷	▷	▷	▷	▷	▷	▷	▷	▷	▷	▷	▷	▷	▷	□	▷	▷	▷	▷	▷	▷	▷	▷	▷
Position	314	317	320	321	323	325	327	329	333	334	336	338	349	351	353	359	362	364	366	367	375	376	385	386

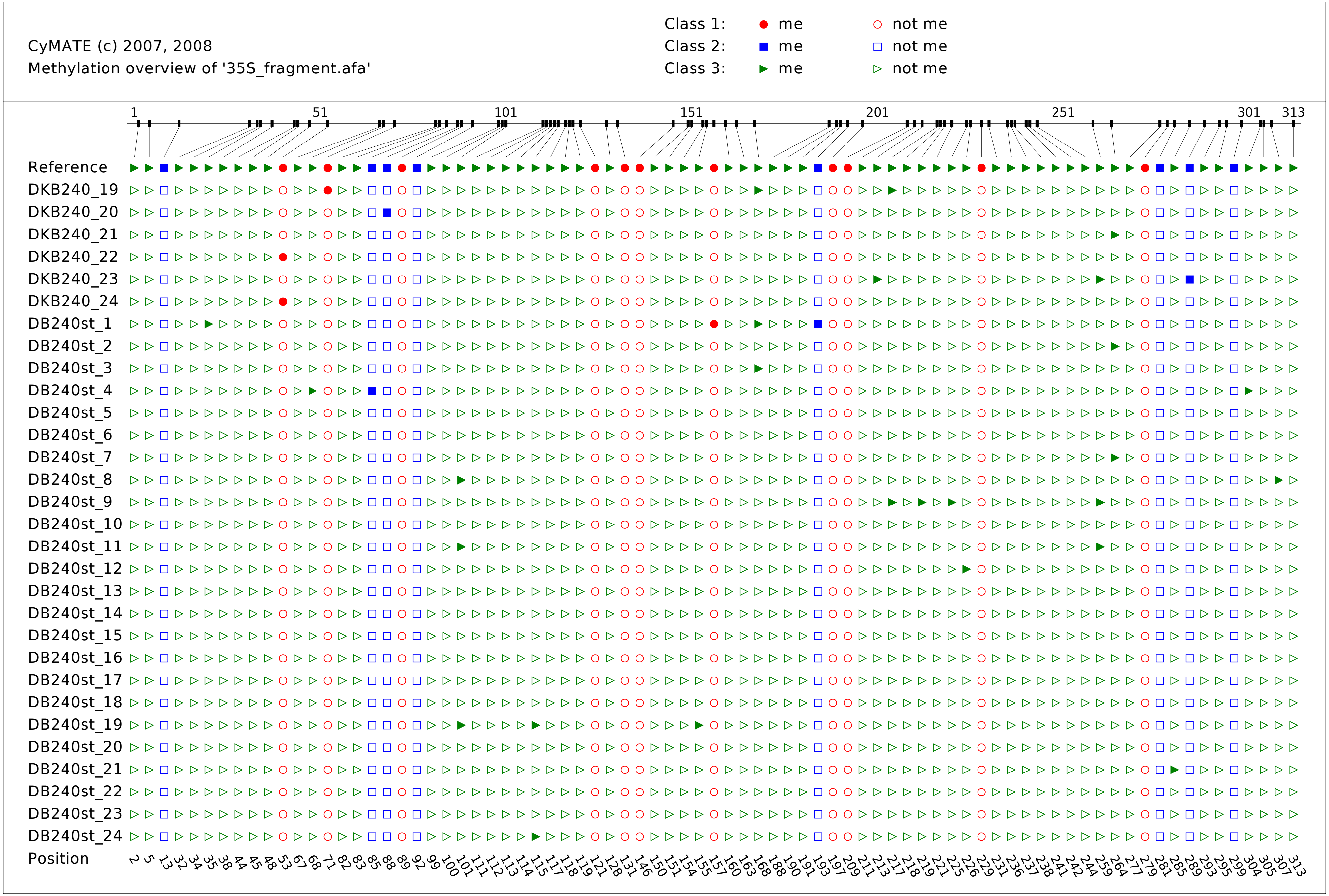
CyMATE (c) 2007, 2008
Methylation overview of '35S_fragment.afa'
Class 1: me not me
Class 2: me not me
Class 3: me not me
1 51 101 151 201 251 301 313
Reference
DKB240_19
DKB240_20
DKB240_21
DKB240_22
DKB240_23
DKB240_24
DB240st_1
DB240st_2
DB240st_3
DB240st_4
DB240st_5
DB240st_6
DB240st_7
DB240st_8
DB240st_9
DB240st_10
DB240st_11
DB240st_12
DB240st_13
DB240st_14
DB240st_15
DB240st_16
DB240st_17
DB240st_18
DB240st_19
DB240st_20
DB240st_21
DB240st_22
DB240st_23
DB240st_24
Position
2 5 13 32 34 35 38 44 45 48 53 67 68 71 82 83 85 88 89 92 99 100 101 111 112 113 114 115 117 118 119 121 128 131 146 150 151 154 155 157 160 163 168 188 190 191 193 197 209 211 213 217 218 219 221 225 226 229 231 236 237 238 241 242 244 259 264 277 279 281 285 289 293 295 299 304 305 307 313

CyMATE (c) 2007, 2008
Methylation overview of '35S_fragment.afa'

Class 1: ● me ○ not me
Class 2: ■ me □ not me
Class 3: ▶ me ▷ not me

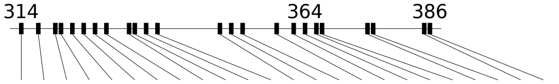

Position	314	317	320	321	323	325	327	329	333	334	336	338	349	351	353	359	362	364	366	367	375	376	385	386
Reference	▶	▶	▶	▶	▶	▶	▶	▶	▶	▶	▶	▶	▶	▶	■	▶	▶	▶	▶	▶	▶	▶	▶	▶
DKB240_19	▷	▷	▷	▷	▷	▷	▷	▷	▷	▷	▷	▷	▷	▷	□	▷	▷	▷	▷	▷	▷	▷	▷	▷
DKB240_20	▷	▷	▷	▷	▷	▷	▷	▷	▷	▷	▷	▷	▷	▷	□	▷	▷	▷	▷	▷	▷	▷	▷	▷
DKB240_21	▷	▷	▷	▷	▷	▷	▷	▷	▷	▷	▷	▷	▷	▷	□	▷	▷	▷	▷	▷	▷	▷	▷	▷
DKB240_22	▷	▷	▷	▷	▷	▷	▷	▷	▷	▷	▷	▷	▷	▷	□	▷	▷	▷	▷	▷	▷	▷	▷	▷
DKB240_23	▷	▷	▷	▷	▶	▷	▷	▷	▷	▷	▷	▷	▷	▷	□	▷	▷	▷	▷	▷	▷	▷	▷	▷
DKB240_24	▷	▷	▷	▷	▷	▷	▷	▷	▷	▷	▷	▷	▷	▷	□	▷	▷	▷	▶	▷	▷	▷	▷	▷
DB240st_1	▷	▷	▷	▷	▷	▷	▷	▷	▷	▷	▶	▷	▷	▷	□	▷	▷	▷	▷	▷	▷	▷	▷	▷
DB240st_2	▷	▷	▷	▷	▷	▷	▷	▷	▷	▷	▷	▷	▷	▷	□	▷	▷	▷	▷	▷	▷	▷	▷	▷
DB240st_3	▷	▷	▷	▷	▷	▷	▷	▷	▷	▷	▷	▷	▷	▷	□	▷	▷	▷	▷	▷	▷	▷	▷	▷
DB240st_4	▷	▷	▷	▷	▷	▷	▷	▶	▷	▷	▷	▷	▷	▷	□	▷	▶	▷	▷	▷	▷	▷	▷	▷
DB240st_5	▷	▷	▷	▷	▷	▷	▷	▷	▶	▷	▷	▷	▷	▷	□	▷	▷	▷	▷	▷	▷	▷	▷	▷
DB240st_6	▷	▷	▷	▷	▷	▷	▷	▷	▶	▷	▷	▷	▷	▷	□	▷	▷	▷	▷	▷	▷	▷	▷	▷
DB240st_7	▷	▷	▷	▷	▷	▷	▷	▷	▷	▷	▷	▷	▷	▷	□	▷	▷	▷	▷	▷	▷	▷	▷	▷
DB240st_8	▷	▷	▷	▷	▷	▷	▷	▷	▷	▷	▷	▷	▷	▶	□	▷	▷	▷	▷	▷	▷	▷	▷	▷
DB240st_9	▷	▷	▶	▶	▷	▷	▷	▷	▷	▷	▷	▷	▷	▷	□	▷	▷	▷	▷	▷	▷	▷	▷	▷
DB240st_10	▷	▷	▷	▷	▷	▷	▷	▷	▷	▷	▷	▷	▷	▷	□	▷	▷	▷	▷	▷	▷	▷	▷	▷
DB240st_11	▷	▷	▷	▷	▷	▷	▷	▷	▷	▷	▷	▷	▷	▷	□	▷	▷	▷	▷	▷	▷	▷	▷	▷
DB240st_12	▷	▷	▷	▷	▷	▷	▷	▷	▷	▷	▶	▷	▷	▷	□	▷	▷	▷	▷	▷	▷	▷	▷	▷
DB240st_13	▷	▷	▷	▷	▷	▷	▷	▷	▷	▷	▷	▷	▷	▷	□	▷	▷	▷	▷	▷	▷	▷	▷	▷
DB240st_14	▷	▷	▷	▷	▷	▷	▷	▷	▷	▷	▷	▷	▷	▷	□	▷	▷	▷	▷	▷	▷	▷	▷	▷
DB240st_15	▷	▷	▷	▷	▶	▷	▷	▷	▷	▷	▷	▷	▷	▷	□	▷	▷	▷	▷	▷	▷	▷	▷	▷
DB240st_16	▷	▷	▷	▷	▷	▶	▷	▷	▷	▷	▷	▷	▷	▷	□	▷	▷	▷	▷	▷	▷	▷	▷	▷
DB240st_17	▷	▷	▷	▷	▷	▷	▷	▷	▷	▷	▷	▷	▷	▷	□	▷	▷	▷	▷	▷	▷	▷	▷	▷
DB240st_18	▷	▷	▷	▷	▷	▷	▷	▷	▷	▷	▷	▷	▷	▷	□	▷	▷	▷	▷	▷	▷	▷	▷	▷
DB240st_19	▷	▷	▷	▷	▷	▷	▷	▷	▷	▷	▷	▷	▷	▶	□	▷	▷	▷	▷	▷	▷	▷	▷	▷
DB240st_20	▷	▷	▷	▷	▷	▷	▷	▷	▷	▷	▷	▷	▷	▷	□	▷	▷	▷	▷	▷	▷	▷	▷	▷
DB240st_21	▷	▷	▷	▷	▷	▷	▷	▷	▷	▷	▷	▷	▷	▷	□	▷	▷	▷	▷	▷	▷	▷	▷	▷
DB240st_22	▶	▷	▷	▷	▷	▷	▷	▷	▷	▷	▷	▷	▶	▷	□	▷	▷	▷	▷	▷	▷	▷	▷	▷
DB240st_23	▷	▷	▷	▷	▷	▷	▷	▷	▷	▷	▷	▷	▷	▷	□	▷	▷	▷	▷	▷	▷	▷	▷	▷
DB240st_24	▷	▷	▷	▷	▷	▷	▷	▷	▷	▷	▷	▷	▷	▷	□	▷	▷	▷	▷	▷	▷	▷	▷	▷
