# Supplementary material for: Levels of DNA methylation and transcript accumulation in leaves of transgenic maize varieties
Source: Environ Sci Eur. 2016 Nov 23;28(1):29. doi: 10.1186/s12302-016-0097-2 (PMC5120055; doi:10.1186/s12302-016-0097-2)

CyMATE (c) 2007, 2008

Methylation overview of 'cry1A.105_fragment.afa'

Class 1: ● me ○ not me

Class 2: ■ me □ not me

Class 3: ▶ me ▷ not me

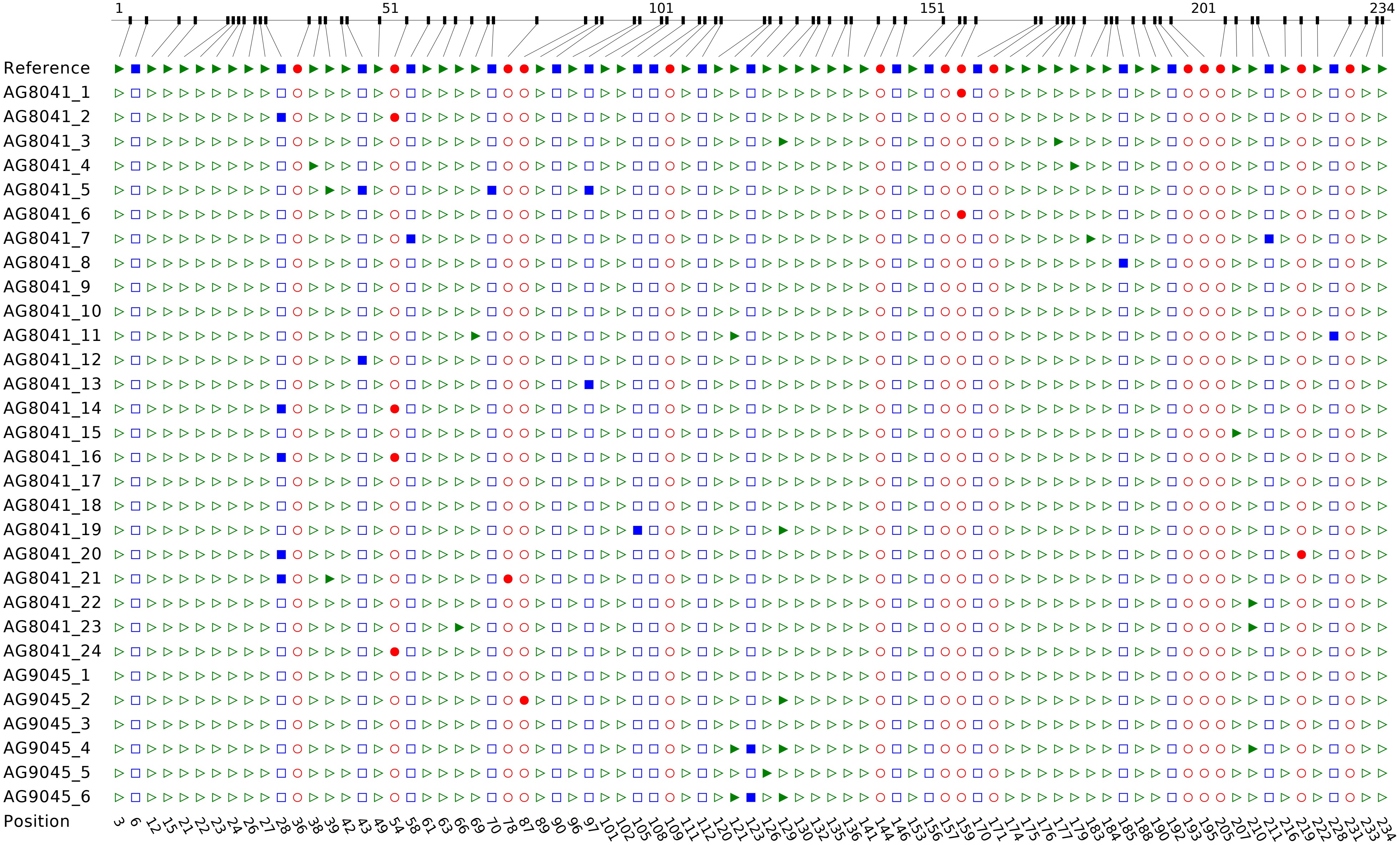

CyMATE (c) 2007, 2008
Methylation overview of 'cry1A.105_fragment.afa'

Class 1: ● me ○ not me
Class 2: ■ me □ not me
Class 3: ▶ me ▷ not me

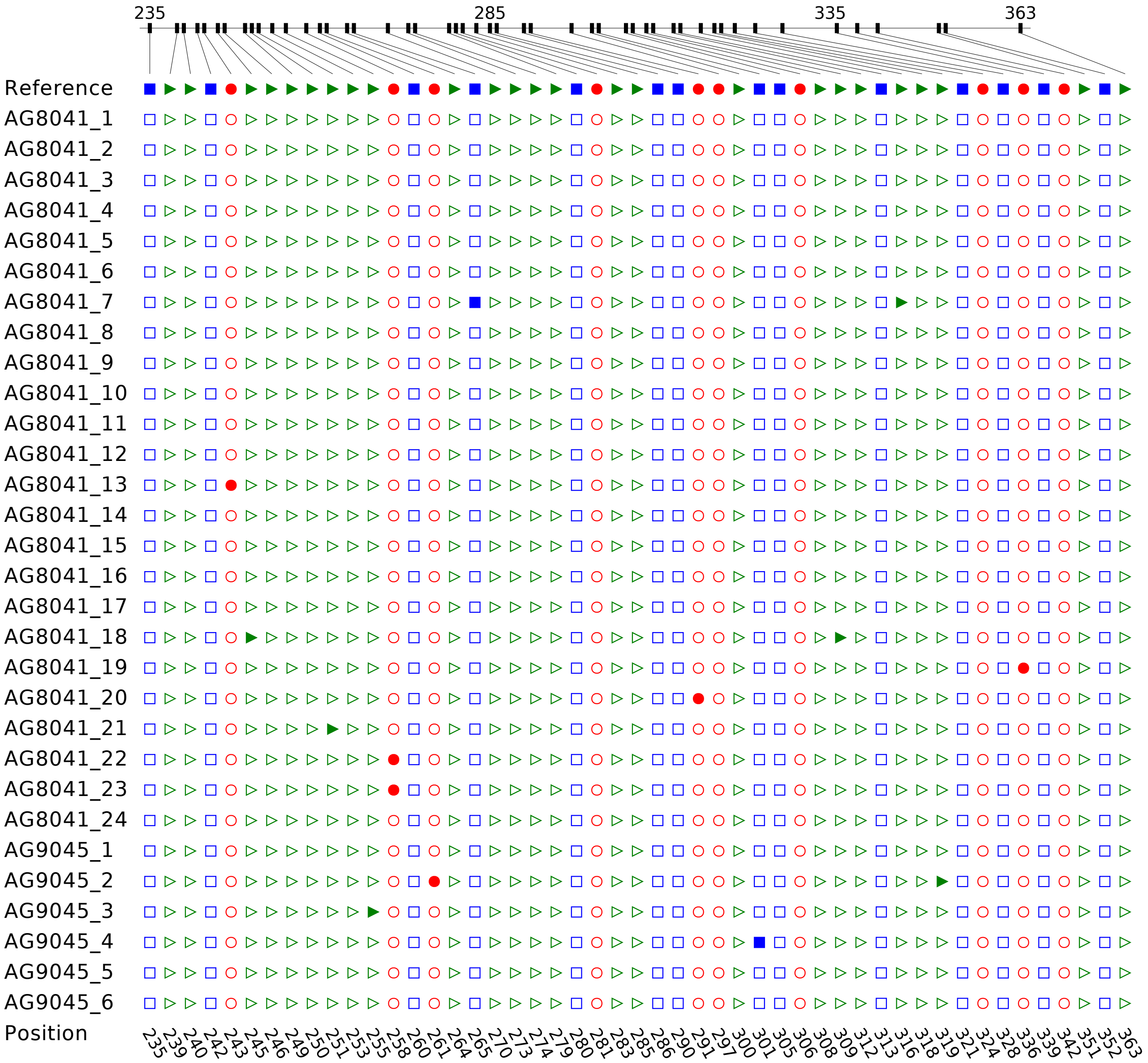

CyMATE (c) 2007, 2008

Methylation overview of 'cry1A.105_fragment.afa'

Class 1: ● me ○ not me

Class 2: ■ me □ not me

Class 3: ▶ me ▷ not me

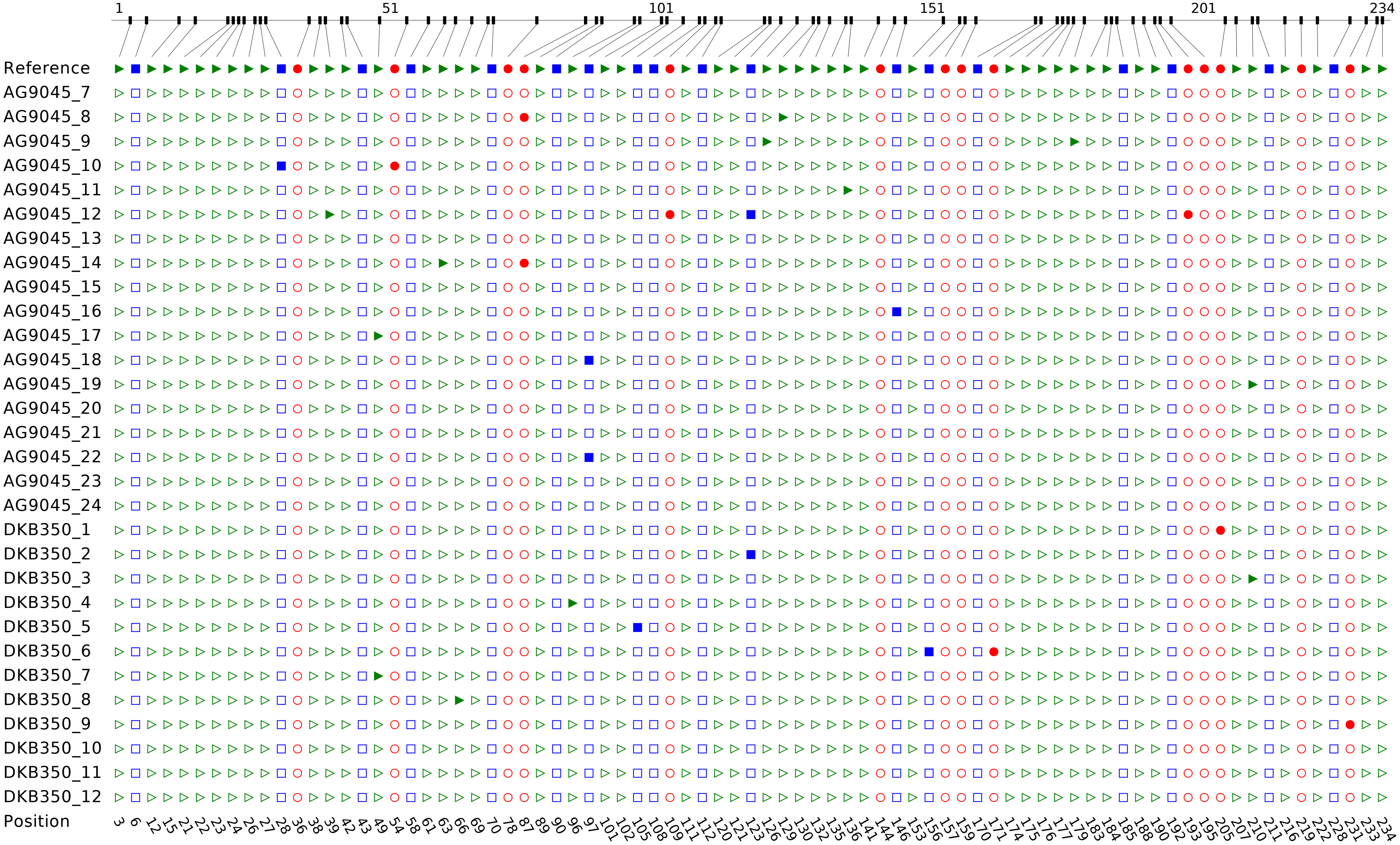

CyMATE (c) 2007, 2008
Methylation overview of 'cry1A.105_fragment.afa'

Class 1: ● me ○ not me
Class 2: ■ me □ not me
Class 3: ▶ me ▷ not me

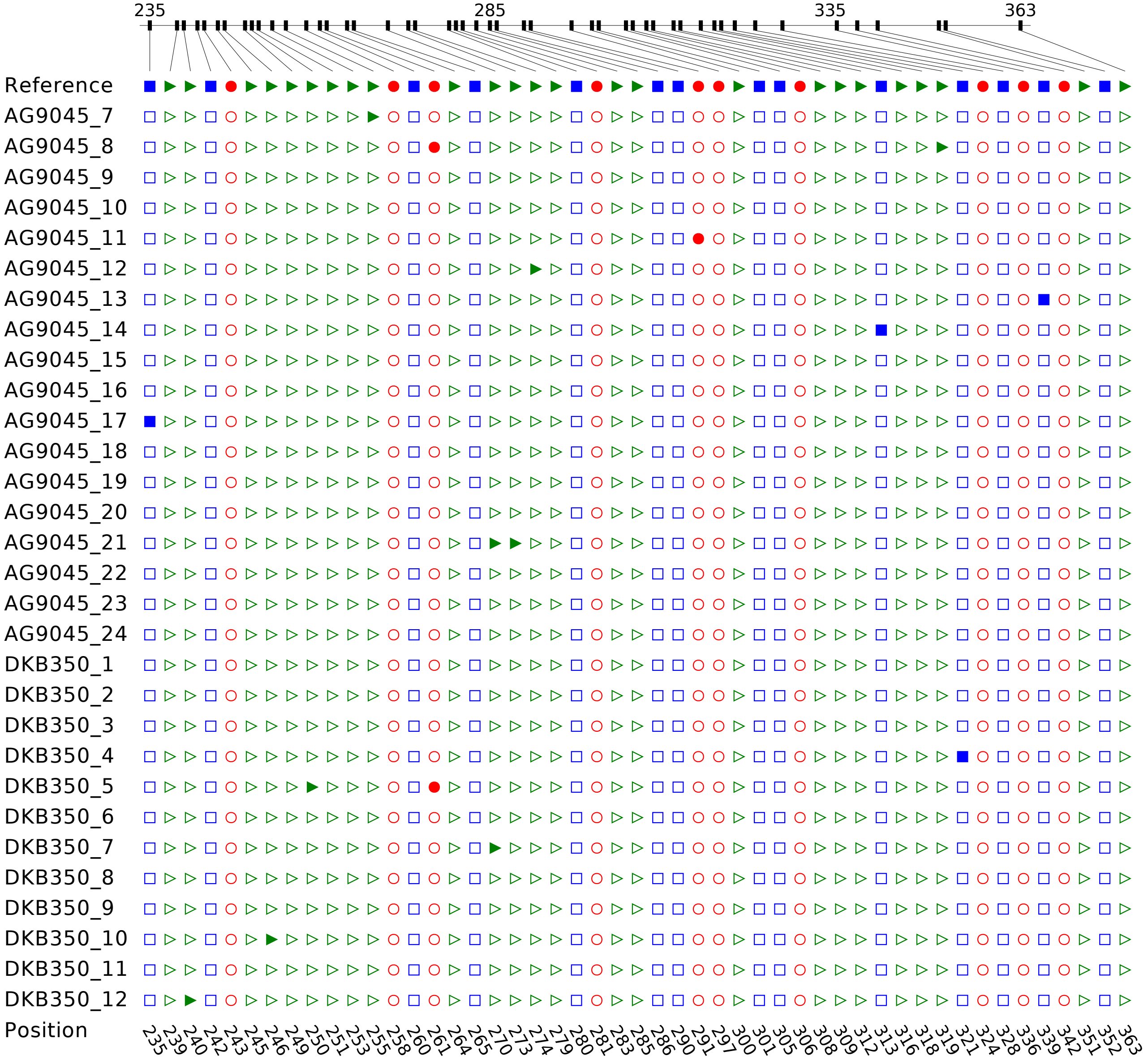

CyMATE (c) 2007, 2008
Methylation overview of 'cry1A.105_fragment.afa'

Class 1: ● me ○ not me
Class 2: ■ me □ not me
Class 3: ▶ me ▷ not me

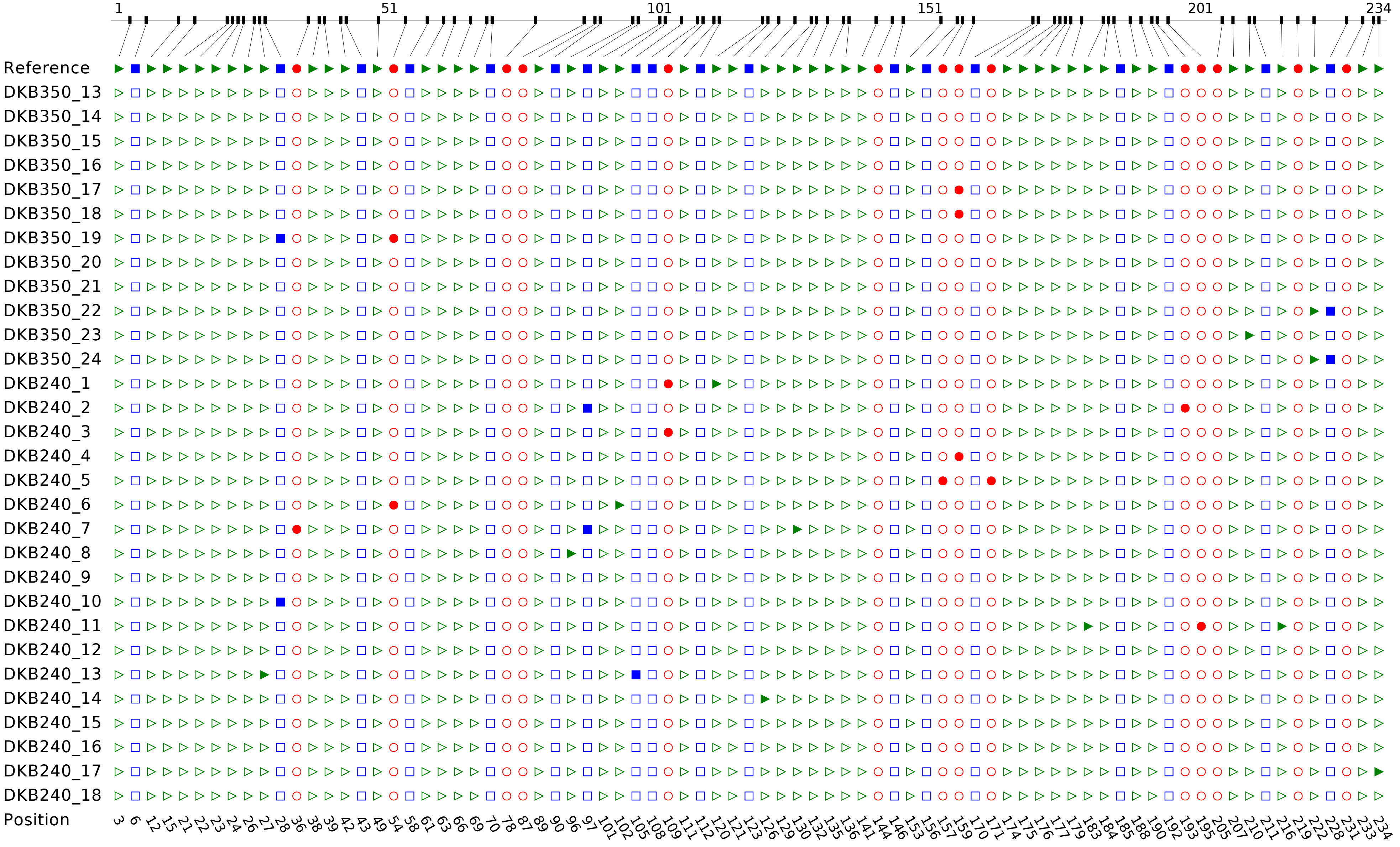

CyMATE (c) 2007, 2008
Methylation overview of 'cry1A.105_fragment.afa'

Class 1: ● me ○ not me
Class 2: ■ me □ not me
Class 3: ▶ me ▷ not me

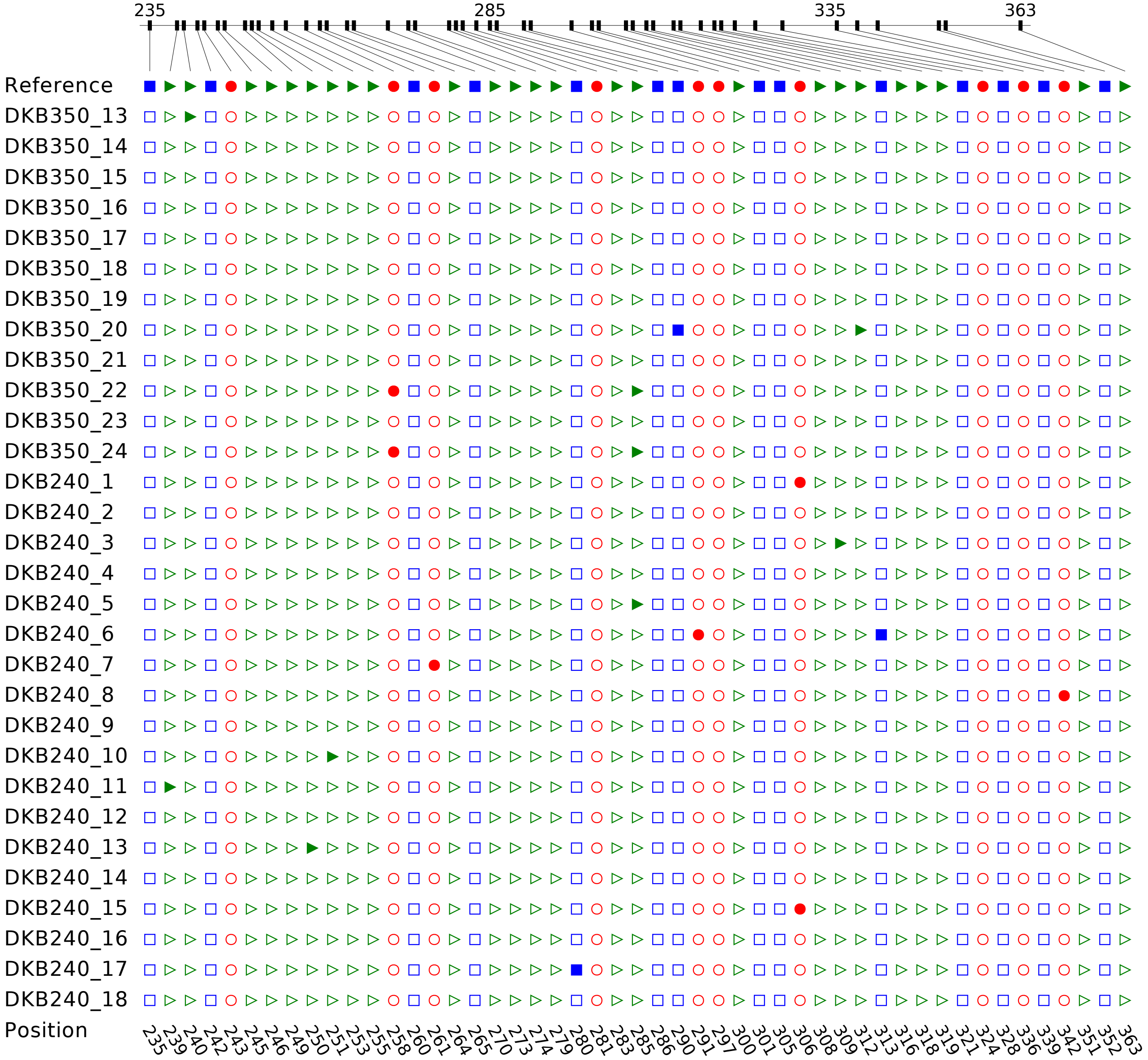

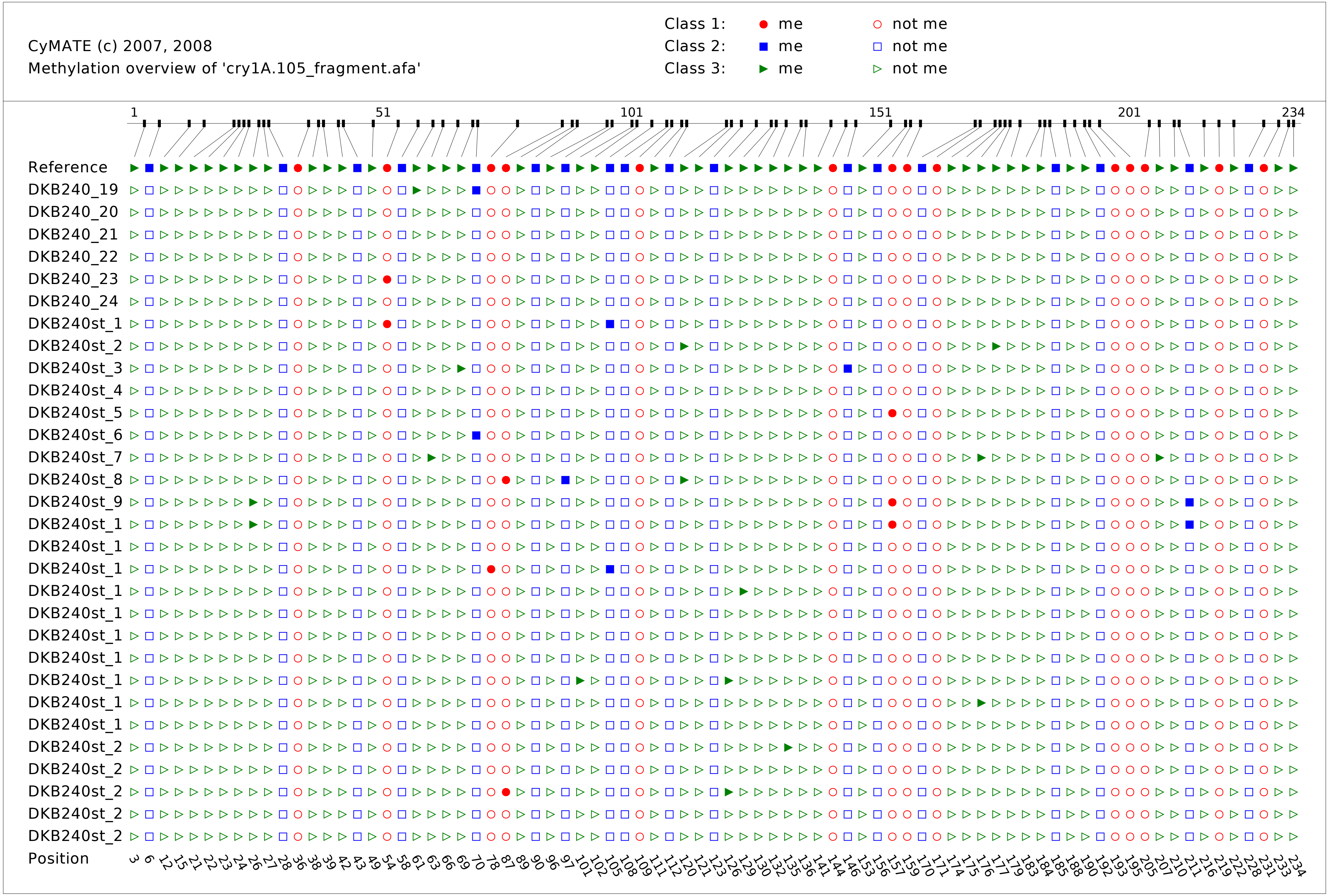
CyMATE (c) 2007, 2008
Methylation overview of 'cry1A.105_fragment.afa'
Class 1: me not me
Class 2: me not me
Class 3: me not me
1
51
101
151
201
234
Reference
DKB240_19
DKB240_20
DKB240_21
DKB240_22
DKB240_23
DKB240_24
DKB240st_1
DKB240st_2
DKB240st_3
DKB240st_4
DKB240st_5
DKB240st_6
DKB240st_7
DKB240st_8
DKB240st_9
DKB240st_1
DKB240st_1
DKB240st_1
DKB240st_1
DKB240st_1
DKB240st_1
DKB240st_1
DKB240st_1
DKB240st_1
DKB240st_1
DKB240st_2
DKB240st_2
DKB240st_2
DKB240st_2
DKB240st_2
Position
3 6 12 15 21 22 23 24 26 27 28 36 38 39 42 43 49 54 58 61 63 66 69 70 78 87 89 90 96 97 101 102 105 108 109 111 112 120 121 123 126 129 130 132 135 136 141 144 146 153 156 157 159 170 171 174 175 176 177 179 183 184 185 188 190 192 193 195 205 207 210 211 216 219 222 228 231 233 234

CyMATE (c) 2007, 2008
Methylation overview of 'cry1A.105_fragment.afa'

Class 1: ● me ○ not me
Class 2: ■ me □ not me
Class 3: ▶ me ▷ not me

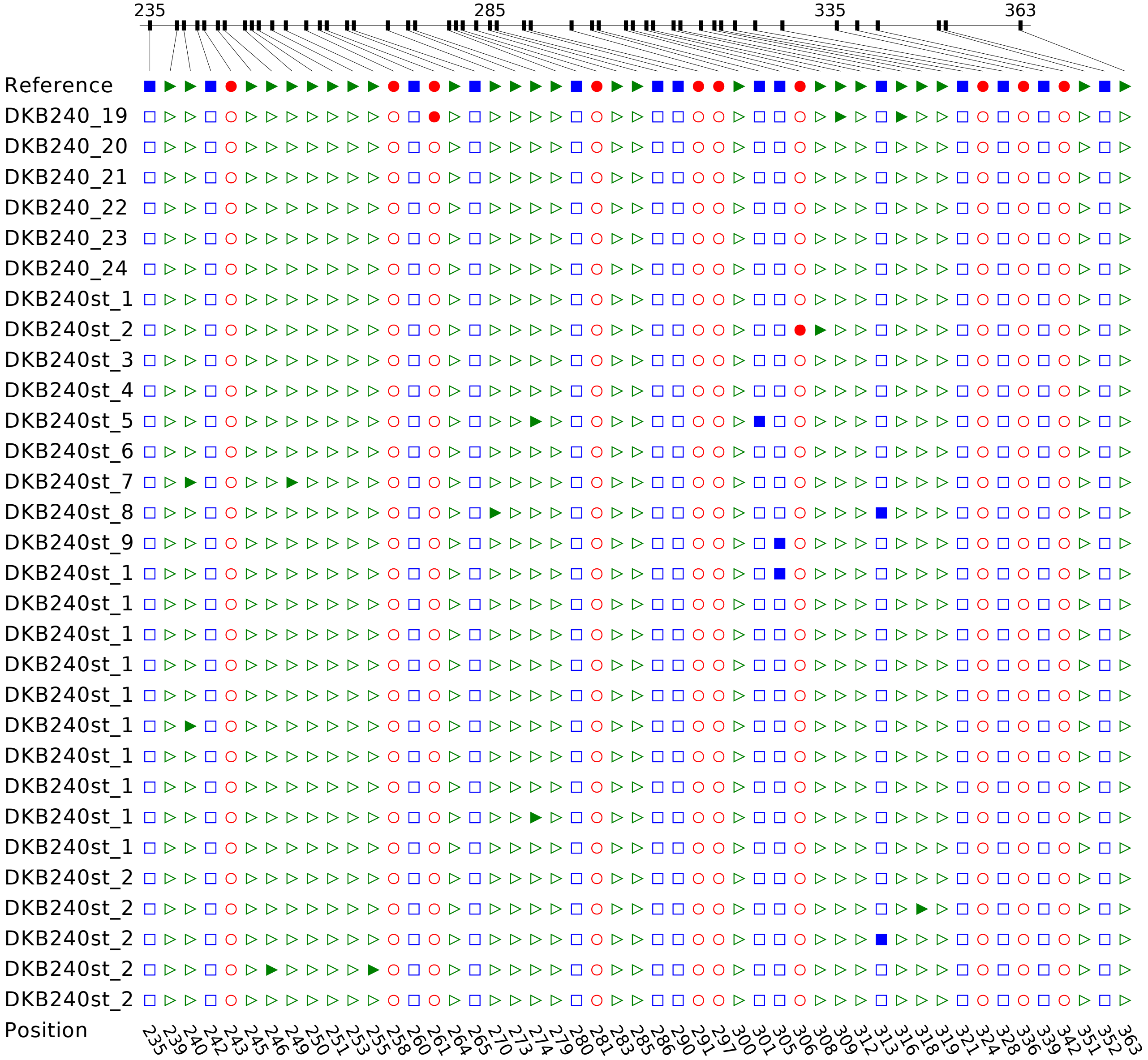

Supplement: Supplementary file 4 — Additional file 4. Position and distribution of methylated and unmethylated cytosines for the cry1A.105 gene sequence in all analysed hybrids. Each line corresponds to one sequenced bacteria colony (clone). Clones from the same hybrid contain the same nomenclature, ranging from 1–24. Class I: CG residue; Class II: CHG residue; Class III: CHH residue. Filled icons: methylated cytosine; Empty icons: non-methylated cytosines. [file 12302_2016_97_MOESM4_ESM.pdf]
