# Supplementary material for: Levels of DNA methylation and transcript accumulation in leaves of transgenic maize varieties
Source: Environ Sci Eur. 2016 Nov 23;28(1):29. doi: 10.1186/s12302-016-0097-2 (PMC5120055; doi:10.1186/s12302-016-0097-2)
Supplement: Supplementary file 5 — Additional file 5. Position and distribution of methylated and unmethylated cytosines for the FMV promoter sequence in all analysed hybrids. Each line corresponds to one sequenced bacteria colony (clone). Clones from the same hybrid contain the same nomenclature, ranging from 1–24. Class I: CG residue; Class II: CHG residue; Class III: CHH residue. Filled icons: methylated cytosine; Empty icons: non-methylated cytosines. [file 12302_2016_97_MOESM5_ESM.pdf]

CyMATE (c) 2007, 2008

Methylation overview of 'FMV_fragment.afa'

Class 1: ● me ○ not me

Class 2: ■ me □ not me

Class 3: ▶ me ▷ not me

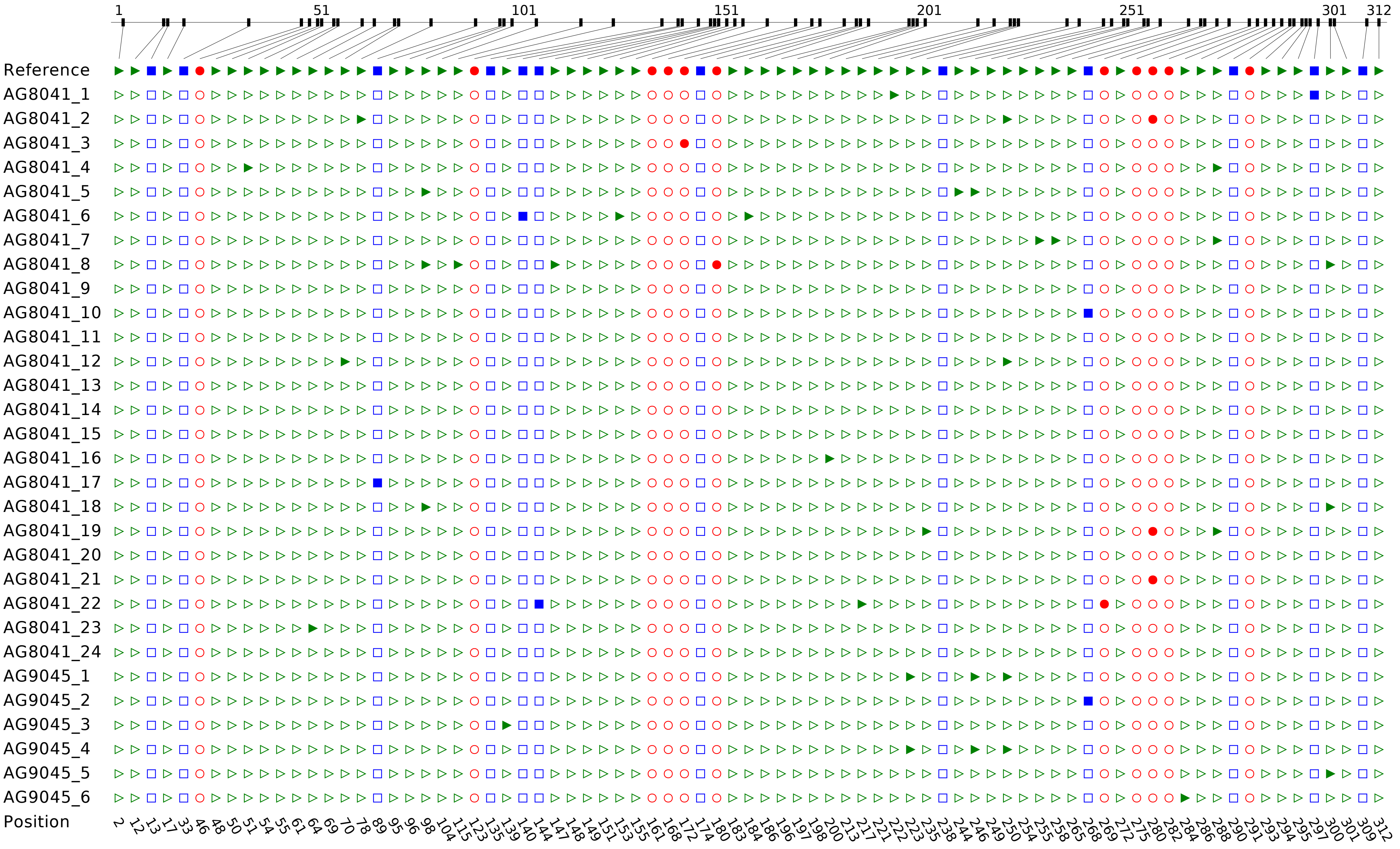

CyMATE (c) 2007, 2008
Methylation overview of 'FMV_fragment.afa'

| | | |
|---|---|---|
| Class 1: | ● me | ○ not me |
| Class 2: | ■ me | □ not me |
| Class 3: | ▶ me | ▷ not me |

313 333

| | | | | | | | |
|---|---|---|---|---|---|---|---|
| Reference | ▶ | ● | ▶ | ■ | ▶ | ▶ | ▶ |
| AG8041_1 | ▶ | ○ | ▷ | □ | ▷ | ▷ | ▷ |
| AG8041_2 | ▷ | ○ | ▷ | □ | ▷ | ▷ | ▷ |
| AG8041_3 | ▷ | ○ | ▷ | □ | ▷ | ▷ | ▷ |
| AG8041_4 | ▷ | ○ | ▷ | □ | ▷ | ▷ | ▷ |
| AG8041_5 | ▷ | ○ | ▷ | □ | ▷ | ▷ | ▷ |
| AG8041_6 | ▷ | ○ | ▷ | □ | ▷ | ▷ | ▷ |
| AG8041_7 | ▷ | ○ | ▷ | □ | ▷ | ▷ | ▷ |
| AG8041_8 | ▷ | ○ | ▷ | □ | ▷ | ▷ | ▷ |
| AG8041_9 | ▷ | ○ | ▷ | □ | ▷ | ▷ | ▷ |
| AG8041_10 | ▷ | ○ | ▷ | □ | ▷ | ▷ | ▷ |
| AG8041_11 | ▷ | ○ | ▷ | □ | ▷ | ▷ | ▷ |
| AG8041_12 | ▷ | ○ | ▷ | □ | ▷ | ▷ | ▷ |
| AG8041_13 | ▷ | ○ | ▷ | □ | ▷ | ▷ | ▷ |
| AG8041_14 | ▷ | ○ | ▷ | □ | ▷ | ▷ | ▷ |
| AG8041_15 | ▷ | ○ | ▷ | □ | ▷ | ▷ | ▷ |
| AG8041_16 | ▷ | ○ | ▷ | □ | ▷ | ▷ | ▷ |
| AG8041_17 | ▷ | ○ | ▷ | □ | ▷ | ▷ | ▷ |
| AG8041_18 | ▷ | ○ | ▷ | □ | ▷ | ▷ | ▷ |
| AG8041_19 | ▷ | ○ | ▷ | □ | ▷ | ▷ | ▷ |
| AG8041_20 | ▷ | ○ | ▷ | □ | ▷ | ▷ | ▷ |
| AG8041_21 | ▷ | ○ | ▷ | □ | ▷ | ▷ | ▷ |
| AG8041_22 | ▷ | ○ | ▷ | □ | ▷ | ▷ | ▷ |
| AG8041_23 | ▷ | ○ | ▷ | □ | ▷ | ▷ | ▷ |
| AG8041_24 | ▷ | ○ | ▷ | □ | ▷ | ▷ | ▷ |
| AG9045_1 | ▷ | ○ | ▷ | □ | ▷ | ▷ | ▷ |
| AG9045_2 | ▷ | ○ | ▷ | □ | ▷ | ▷ | ▷ |
| AG9045_3 | ▷ | ○ | ▷ | □ | ▷ | ▷ | ▷ |
| AG9045_4 | ▷ | ○ | ▷ | □ | ▷ | ▷ | ▷ |
| AG9045_5 | ▷ | ○ | ▷ | □ | ▷ | ▷ | ▷ |
| AG9045_6 | ▷ | ○ | ▷ | □ | ▷ | ▷ | ▷ |
| Position | 313 | 315 | 320 | 322 | 329 | 331 | 333 |

CyMATE (c) 2007, 2008

Methylation overview of 'FMV_fragment.afa'

Class 1: ● me ○ not me

Class 2: ■ me □ not me

Class 3: ▶ me ▷ not me

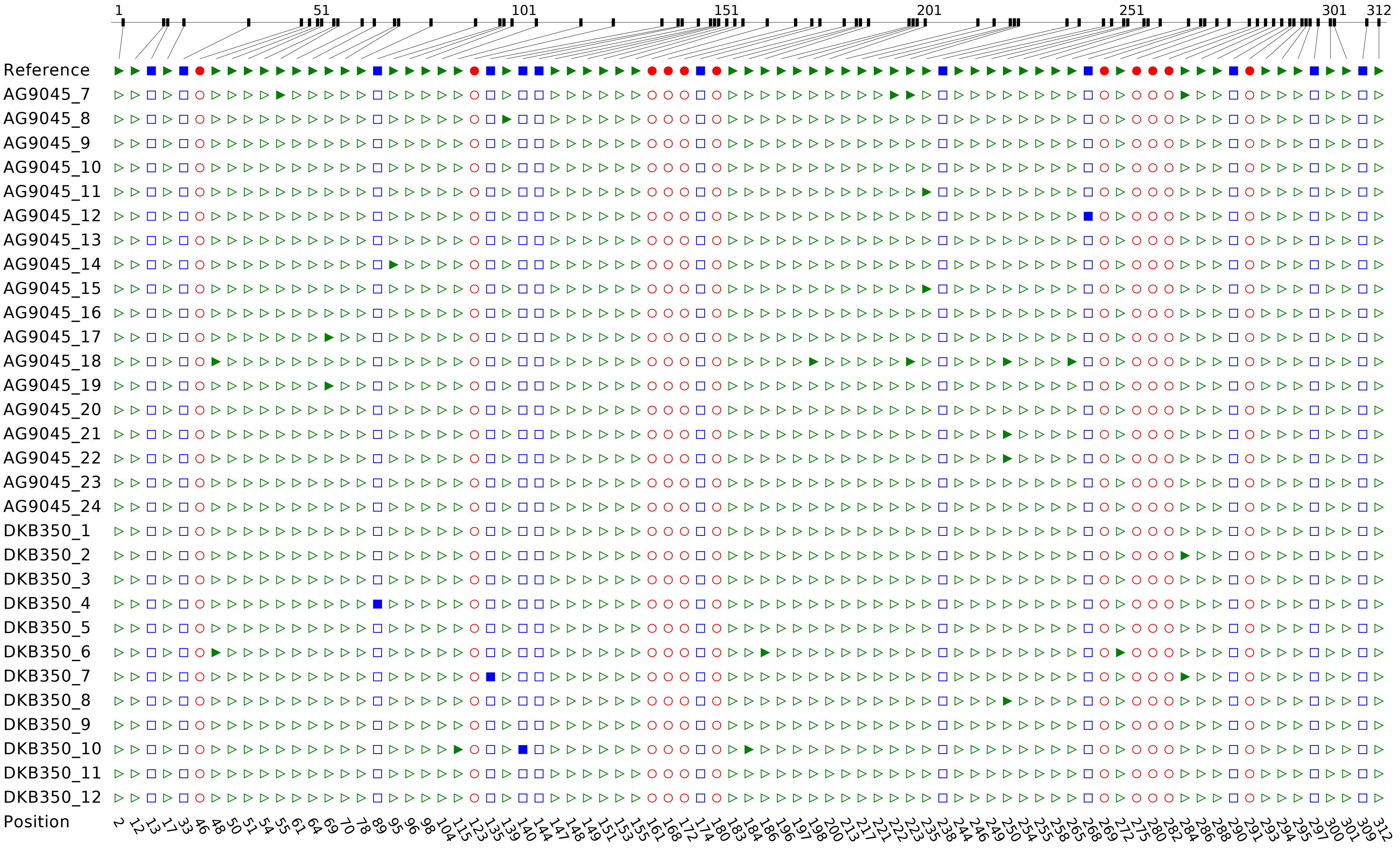

CyMATE (c) 2007, 2008
Methylation overview of 'FMV_fragment.afa'

| | | | |
|---|---|---|---|
| Class 1: | ● | me | ○ not me |
| Class 2: | ■ | me | □ not me |
| Class 3: | ▶ | me | ▷ not me |

| Position | 313 | 315 | 320 | 322 | 329 | 331 | 333 |
|---|---|---|---|---|---|---|---|
| Reference | ▶ | ● | ▶ | ■ | ▶ | ▶ | ▶ |
| AG9045_7 | ▷ | ○ | ▷ | □ | ▷ | ▷ | ▷ |
| AG9045_8 | ▷ | ○ | ▷ | □ | ▷ | ▷ | ▷ |
| AG9045_9 | ▷ | ○ | ▷ | □ | ▷ | ▷ | ▷ |
| AG9045_10 | ▷ | ○ | ▷ | □ | ▷ | ▷ | ▷ |
| AG9045_11 | ▷ | ○ | ▷ | □ | ▷ | ▷ | ▷ |
| AG9045_12 | ▷ | ○ | ▷ | □ | ▷ | ▷ | ▷ |
| AG9045_13 | ▷ | ○ | ▷ | □ | ▷ | ▷ | ▷ |
| AG9045_14 | ▷ | ○ | ▷ | □ | ▷ | ▷ | ▷ |
| AG9045_15 | ▷ | ○ | ▷ | □ | ▷ | ▷ | ▷ |
| AG9045_16 | ▷ | ○ | ▷ | □ | ▷ | ▷ | ▷ |
| AG9045_17 | ▷ | ○ | ▷ | □ | ▷ | ▷ | ▷ |
| AG9045_18 | ▷ | ○ | ▷ | □ | ▷ | ▷ | ▷ |
| AG9045_19 | ▷ | ○ | ▷ | □ | ▷ | ▷ | ▷ |
| AG9045_20 | ▷ | ○ | ▷ | □ | ▷ | ▷ | ▷ |
| AG9045_21 | ▷ | ○ | ▷ | □ | ▷ | ▷ | ▷ |
| AG9045_22 | ▷ | ○ | ▷ | □ | ▷ | ▷ | ▷ |
| AG9045_23 | ▷ | ○ | ▷ | □ | ▷ | ▷ | ▷ |
| AG9045_24 | ▷ | ○ | ▷ | □ | ▷ | ▷ | ▷ |
| DKB350_1 | ▷ | ○ | ▷ | □ | ▷ | ▷ | ▷ |
| DKB350_2 | ▷ | ○ | ▷ | □ | ▷ | ▷ | ▷ |
| DKB350_3 | ▷ | ○ | ▷ | □ | ▷ | ▷ | ▷ |
| DKB350_4 | ▷ | ○ | ▷ | □ | ▷ | ▷ | ▷ |
| DKB350_5 | ▷ | ○ | ▷ | □ | ▷ | ▷ | ▷ |
| DKB350_6 | ▷ | ○ | ▷ | □ | ▷ | ▷ | ▷ |
| DKB350_7 | ▷ | ○ | ▷ | □ | ▷ | ▷ | ▷ |
| DKB350_8 | ▷ | ○ | ▷ | □ | ▷ | ▷ | ▷ |
| DKB350_9 | ▷ | ○ | ▷ | □ | ▷ | ▷ | ▷ |
| DKB350_10 | ▷ | ○ | ▷ | □ | ▷ | ▷ | ▷ |
| DKB350_11 | ▷ | ○ | ▷ | □ | ▷ | ▷ | ▷ |
| DKB350_12 | ▷ | ○ | ▷ | □ | ▷ | ▷ | ▷ |

CyMATE (c) 2007, 2008

Methylation overview of 'FMV_fragment.afa'

Class 1: ● me ○ not me

Class 2: ■ me □ not me

Class 3: ▶ me ▷ not me

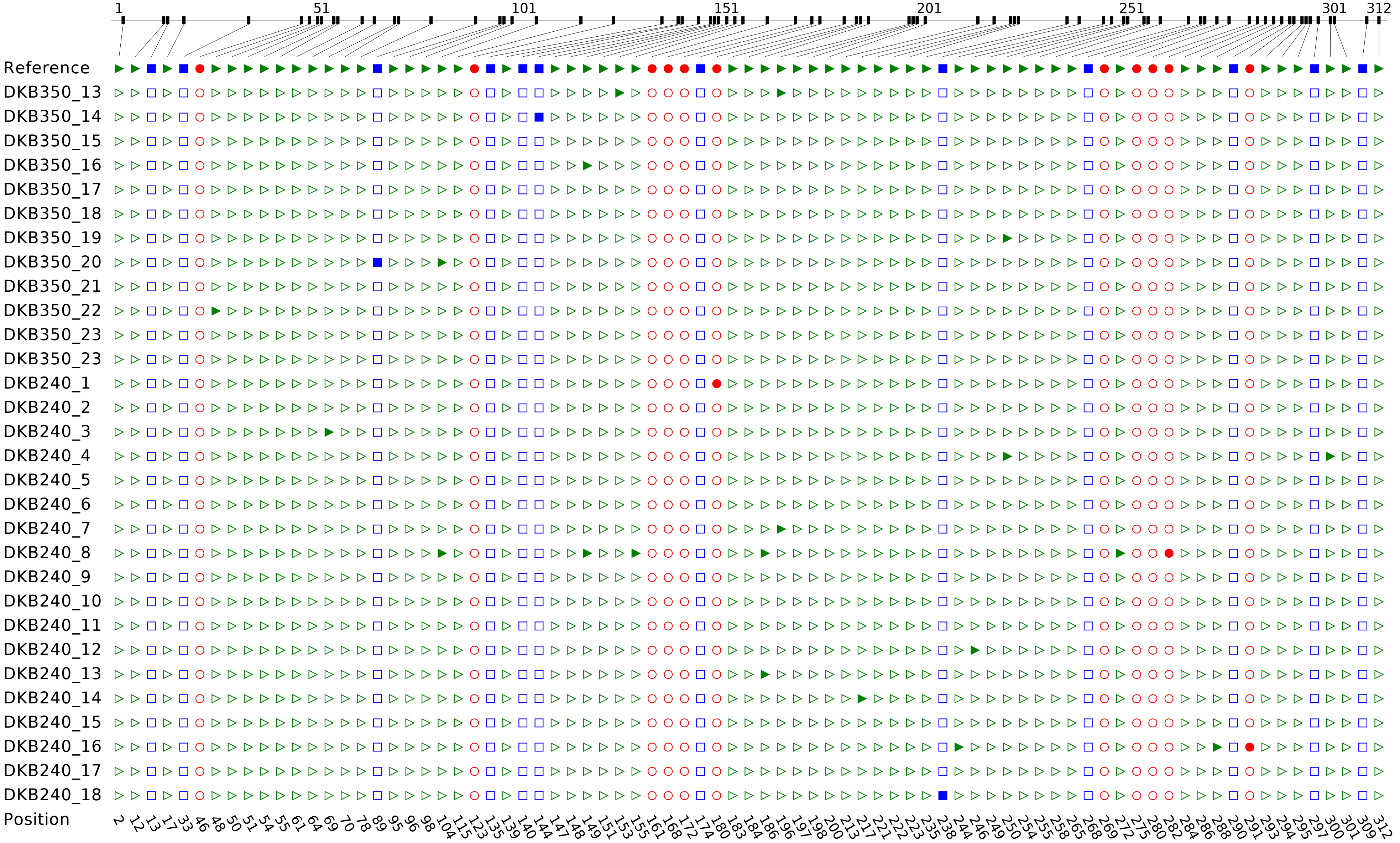

CyMATE (c) 2007, 2008
Methylation overview of 'FMV_fragment.afa'

| | | |
|---|---|---|
| Class 1: | ● me | ○ not me |
| Class 2: | ■ me | □ not me |
| Class 3: | ▶ me | ▷ not me |

| Position | 313 | 315 | 320 | 322 | 329 | 331 | 333 |
|---|---|---|---|---|---|---|---|
| Reference | ▶ | ● | ▶ | ■ | ▶ | ▶ | ▶ |
| DKB350_13 | ▷ | ○ | ▷ | □ | ▷ | ▷ | ▷ |
| DKB350_14 | ▷ | ○ | ▷ | □ | ▷ | ▷ | ▷ |
| DKB350_15 | ▷ | ○ | ▷ | □ | ▷ | ▷ | ▷ |
| DKB350_16 | ▷ | ○ | ▷ | □ | ▷ | ▷ | ▷ |
| DKB350_17 | ▷ | ○ | ▷ | □ | ▷ | ▷ | ▷ |
| DKB350_18 | ▷ | ○ | ▷ | □ | ▷ | ▷ | ▷ |
| DKB350_19 | ▷ | ○ | ▷ | □ | ▷ | ▷ | ▷ |
| DKB350_20 | ▷ | ○ | ▷ | □ | ▷ | ▷ | ▷ |
| DKB350_21 | ▷ | ○ | ▷ | □ | ▷ | ▷ | ▷ |
| DKB350_22 | ▶ | ○ | ▷ | □ | ▷ | ▷ | ▷ |
| DKB350_23 | ▶ | ○ | ▷ | □ | ▷ | ▷ | ▷ |
| DKB350_23 | ▷ | ○ | ▷ | □ | ▷ | ▷ | ▷ |
| DKB240_1 | ▷ | ○ | ▷ | □ | ▷ | ▷ | ▷ |
| DKB240_2 | ▷ | ○ | ▷ | □ | ▷ | ▷ | ▷ |
| DKB240_3 | ▷ | ○ | ▷ | □ | ▷ | ▷ | ▷ |
| DKB240_4 | ▷ | ○ | ▷ | □ | ▷ | ▷ | ▷ |
| DKB240_5 | ▷ | ○ | ▷ | □ | ▷ | ▷ | ▷ |
| DKB240_6 | ▷ | ○ | ▷ | □ | ▷ | ▷ | ▷ |
| DKB240_7 | ▷ | ○ | ▷ | □ | ▷ | ▷ | ▷ |
| DKB240_8 | ▷ | ○ | ▷ | □ | ▷ | ▷ | ▷ |
| DKB240_9 | ▷ | ○ | ▷ | □ | ▷ | ▷ | ▷ |
| DKB240_10 | ▷ | ○ | ▷ | □ | ▷ | ▷ | ▷ |
| DKB240_11 | ▷ | ○ | ▷ | □ | ▷ | ▷ | ▷ |
| DKB240_12 | ▷ | ○ | ▷ | □ | ▷ | ▷ | ▷ |
| DKB240_13 | ▷ | ○ | ▷ | □ | ▷ | ▷ | ▷ |
| DKB240_14 | ▷ | ○ | ▷ | □ | ▷ | ▷ | ▷ |
| DKB240_15 | ▷ | ○ | ▷ | □ | ▷ | ▷ | ▷ |
| DKB240_16 | ▷ | ○ | ▷ | □ | ▷ | ▷ | ▷ |
| DKB240_17 | ▷ | ○ | ▷ | □ | ▷ | ▷ | ▷ |
| DKB240_18 | ▷ | ○ | ▷ | □ | ▷ | ▷ | ▷ |

CyMATE (c) 2007, 2008
Methylation overview of 'FMV_fragment.afa'

Class 1: ● me ○ not me
Class 2: ■ me □ not me
Class 3: ▶ me ▷ not me

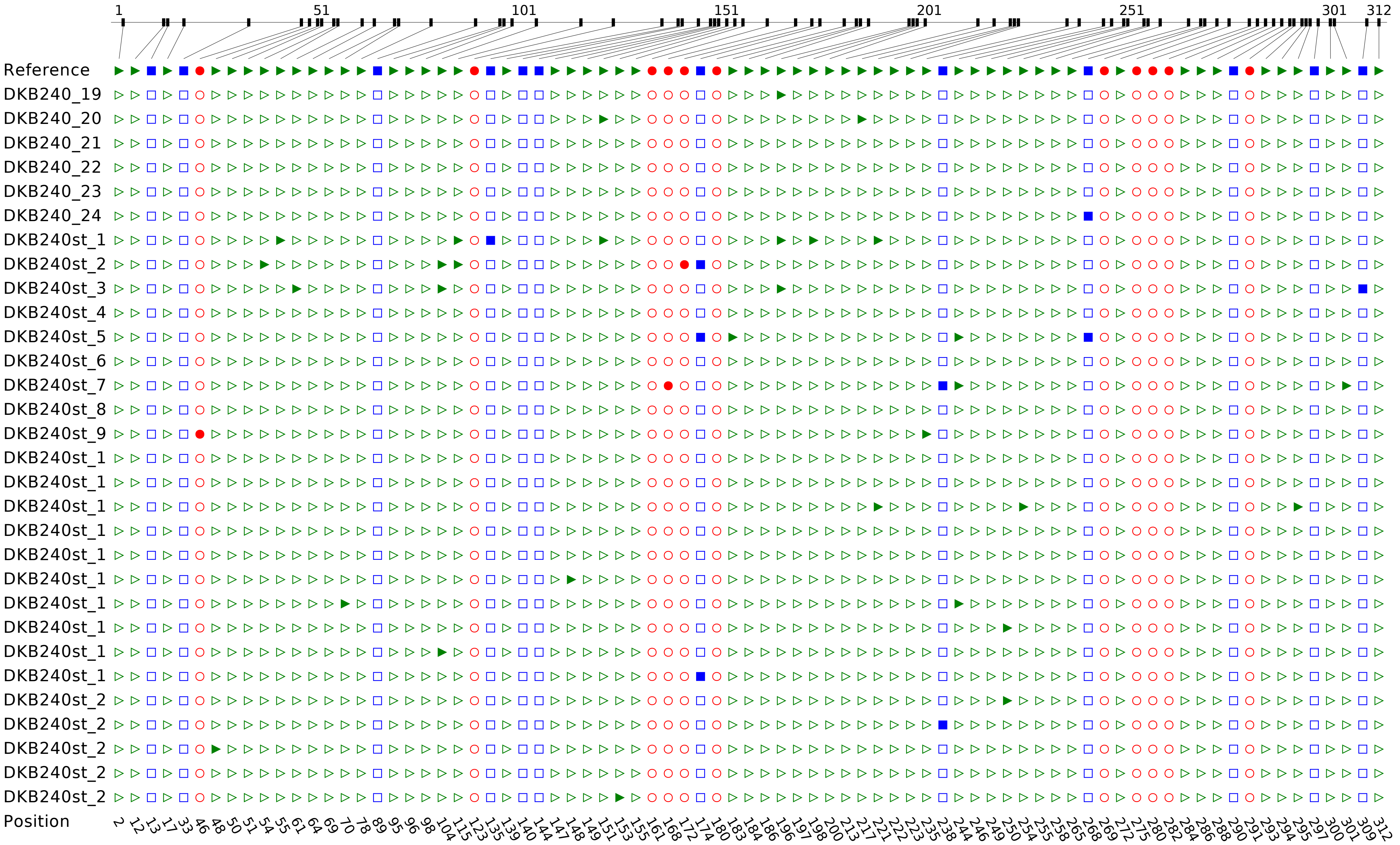

CyMATE (c) 2007, 2008
Methylation overview of 'FMV_fragment.afa'

| | | |
|---|---|---|
| Class 1: | ● me | ○ not me |
| Class 2: | ■ me | □ not me |
| Class 3: | ▶ me | ▷ not me |

313 333

| | | | | | | | |
|---|---|---|---|---|---|---|---|
| Reference | ▶ | ● | ▶ | ■ | ▶ | ▶ | ▶ |
| DKB240_19 | ▷ | ○ | ▷ | □ | ▷ | ▷ | ▷ |
| DKB240_20 | ▷ | ○ | ▷ | □ | ▷ | ▷ | ▷ |
| DKB240_21 | ▷ | ○ | ▷ | □ | ▷ | ▷ | ▷ |
| DKB240_22 | ▷ | ○ | ▷ | □ | ▷ | ▷ | ▷ |
| DKB240_23 | ▷ | ○ | ▶ | □ | ▷ | ▷ | ▷ |
| DKB240_24 | ▷ | ○ | ▷ | □ | ▷ | ▷ | ▷ |
| DKB240st_1 | ▷ | ○ | ▷ | □ | ▷ | ▷ | ▷ |
| DKB240st_2 | ▷ | ○ | ▷ | □ | ▷ | ▷ | ▷ |
| DKB240st_3 | ▷ | ○ | ▷ | □ | ▷ | ▷ | ▷ |
| DKB240st_4 | ▷ | ○ | ▷ | □ | ▷ | ▷ | ▷ |
| DKB240st_5 | ▷ | ○ | ▷ | □ | ▷ | ▷ | ▷ |
| DKB240st_6 | ▷ | ○ | ▷ | □ | ▷ | ▷ | ▷ |
| DKB240st_7 | ▷ | ○ | ▷ | □ | ▷ | ▷ | ▷ |
| DKB240st_8 | ▷ | ○ | ▷ | □ | ▷ | ▷ | ▷ |
| DKB240st_9 | ▷ | ○ | ▷ | □ | ▷ | ▷ | ▷ |
| DKB240st_1 | ▷ | ● | ▷ | □ | ▷ | ▷ | ▷ |
| DKB240st_1 | ▷ | ○ | ▷ | □ | ▷ | ▷ | ▷ |
| DKB240st_1 | ▷ | ○ | ▷ | □ | ▷ | ▷ | ▷ |
| DKB240st_1 | ▷ | ○ | ▷ | □ | ▷ | ▷ | ▷ |
| DKB240st_1 | ▷ | ○ | ▷ | □ | ▷ | ▷ | ▷ |
| DKB240st_1 | ▷ | ○ | ▷ | □ | ▷ | ▷ | ▷ |
| DKB240st_1 | ▷ | ○ | ▷ | □ | ▷ | ▷ | ▷ |
| DKB240st_1 | ▷ | ○ | ▷ | □ | ▷ | ▷ | ▷ |
| DKB240st_1 | ▷ | ○ | ▷ | □ | ▷ | ▷ | ▷ |
| DKB240st_1 | ▷ | ○ | ▷ | □ | ▷ | ▷ | ▷ |
| DKB240st_2 | ▷ | ○ | ▷ | □ | ▷ | ▷ | ▷ |
| DKB240st_2 | ▷ | ○ | ▷ | □ | ▷ | ▷ | ▷ |
| DKB240st_2 | ▷ | ○ | ▷ | □ | ▷ | ▷ | ▷ |
| DKB240st_2 | ▷ | ○ | ▷ | □ | ▷ | ▷ | ▷ |
| DKB240st_2 | ▷ | ○ | ▷ | □ | ▷ | ▷ | ▷ |
| Position | 313 | 315 | 320 | 322 | 329 | 331 | 333 |
